# Targeting Chromatin-Remodeling Factors in Cancer Cells: Promising Molecules in Cancer Therapy

**DOI:** 10.3390/ijms232112815

**Published:** 2022-10-24

**Authors:** Fang-Lin Zhang, Da-Qiang Li

**Affiliations:** 1 Shanghai Cancer Center and Institutes of Biomedical Sciences, Shanghai Medical College, Fudan University, Shanghai 200032, China; 2 Cancer Institute, Shanghai Medical College, Fudan University, Shanghai 200032, China; 3 Department of Oncology, Shanghai Medical College, Fudan University, Shanghai 200032, China; 4 Department of Breast Surgery, Shanghai Medical College, Fudan University, Shanghai 200032, China; 5 Shanghai Key Laboratory of Breast Cancer, Shanghai Medical College, Fudan University, Shanghai 200032, China; 6 Shanghai Key Laboratory of Radiation Oncology, Shanghai Medical College, Fudan University, Shanghai 200032, China

**Keywords:** chromatin remodeling, promising molecules, cancer therapy

## Abstract

ATP-dependent chromatin-remodeling complexes can reorganize and remodel chromatin and thereby act as important regulator in various cellular processes. Based on considerable studies over the past two decades, it has been confirmed that the abnormal function of chromatin remodeling plays a pivotal role in genome reprogramming for oncogenesis in cancer development and/or resistance to cancer therapy. Recently, exciting progress has been made in the identification of genetic alteration in the genes encoding the chromatin-remodeling complexes associated with tumorigenesis, as well as in our understanding of chromatin-remodeling mechanisms in cancer biology. Here, we present preclinical evidence explaining the signaling mechanisms involving the chromatin-remodeling misregulation-induced cancer cellular processes, including DNA damage signaling, metastasis, angiogenesis, immune signaling, etc. However, even though the cumulative evidence in this field provides promising emerging molecules for therapeutic explorations in cancer, more research is needed to assess the clinical roles of these genetic cancer targets.

## 1. Introduction

In eukaryotes, genetic information is stored in the chromatin. Chromatin is organized into repeated units of nucleosomes, in which DNA is tightly packaged into the histone octamer. Two copies of histones (that is the core histones, H2A, H2B, H3, and H4) are linked by histone H1 and comprise the histone octamer, and the assembled histone octamers are further organized to form higher-order chromatin that has several additional chromatin-interacting proteins. Due to compositional diversity, chromatin is highly dynamic and plastic, thereby providing it with high potential to modify genome topology and to orchestrate gene regulation in many aspects of cellular processes [1]. During DNA methylation, the complex post-translational modifications of chromatin proteins and chromatin-remodeling activity are the main heritable epigenetic characteristics [2]. Among these, chromatin remodeling has emerged in recent years as an important regulator for the precise control of the development of tissues and organs, as well as for disease progression in living organisms.

Studies of the underlying mechanistic alterations during disease progression in chromatin remodeling have identified numerous regulatory factors, and have revealed novel mechanistic and functional insights into the relationships of chromatin-remodeling heterogeneity and disease progression, especially in development and treatment of cancer [3]. Chromatin remodeling links the genome with its functional phenotype through several primary mechanisms: (1) ATP-dependent chromatin-remodeling complexes ensure the proper distribution of nucleosomes; (2) remodeling complexes move or eject histones to allow transcription factors to bind to DNA; and (3) remodeling complexes replace the histone with variants of the histone. Thereby, genome-wide nucleosome positioning and composition are tailored by specialized remodelers. In recent years, profound advancements have been made in understanding cancer mechanisms, providing new insights into the molecular processes underlying tumor progression and indicating novel treatment strategies. In addition, the extensively developed molecular biology techniques are allowing a new appreciation of the role of chromatin remodeling in disease development, particularly in cancer [4]. In this review, we provide an overview of our current understanding of chromatin remodeling and its special role in tumor development and treatment, as well as present promising molecules for targeting chromatin-remodeling factors in cancer.

## 2. Dysregulation of Chromatin-Remodeling Machines in Cancer

Chromatin can be either packed in the form of accessible euchromatin, or densely as heterochromatin [5]. Intricately packaged chromatins must be relaxed before the functional complex can be accessed, and the molecular regulatory mechanism of chromatin accessibility is mainly observed through histone modification and ATP-dependent remodelers [6]. Histone enzymes post-translationally modify histone tails and hence alter the atomic structure of nucleosomes to either inhibit or promote the recruitment of various chromatin-associated proteins. So far, several histone modifications have been identified as crucial regulators in cancer progression via controlling chromosomal packing, such as methylation, acetylation, phosphorylation, ADP ribosylation, ubiquitylation, SUMOylating, etc. For example, a histone acetylation-based gene signature was found to be significantly related to the prognosis of ovarian cancer [7]. Histone methylation status can also be marked at specific sites on chromatin, such as transcriptionally repressed regions with a high H3K27me3 signal or in active regions with a rich H3K4me3 signal [8]. It is important to note that aberrant DNA methylation was closely related to cancer development, an example of which is H3K27me3, which was found to play a paramount role in defining the tumor-promoting capacities of cancer-associated fibroblasts [9].

**Figure 1:**
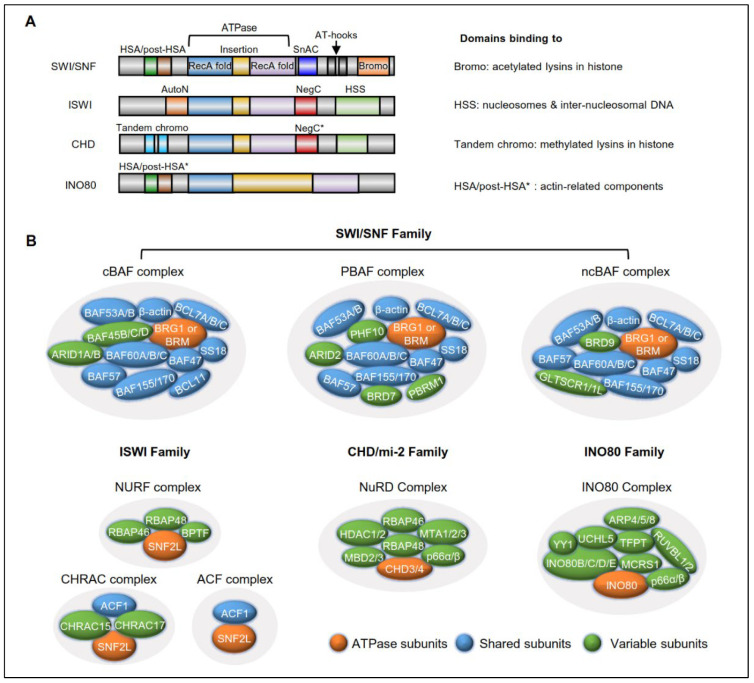
Domain organization and composition of chromatin-remodeling complex. (**A**) Domain organization of four chromatin remodeler complexes. Remodelers can be divided into four subfamilies according to the domain organization in the catalytic ATPases and their associated subunits. The ATPase domains in all of the remodelers are used to mobilize nucleosomes and comprise two RecA-like folds, which are separated by an insertion (yellow). Asterisk represents structural similarity. (**B**) The main complexes in each subfamily. The ATPase subunits, shared subunits, and variable subunits of the representative complexes in each of the four families of human chromatin remodelers.

ATP-dependent remodeling enzymes are other essential mediators of dynamic chromatin and utilize ATP hydrolysis to mobilize nucleosomes, thereby mediating the chromatin structure and the regulation of gene expression [3]. According to the homology in the catalytic ATPases and associated subunits, ATP-dependent chromatin-remodeling complexes can be divided into four subfamilies: switch/sucrose non-fermentable (SWI/SNF), imitation switch (ISWI), chromodomain helicase DNA-binding (CHD) and inositol 80 (INO80) (Figure 1A). The SWI/SNF complex contains a central ATPase domain that includes two RecA-like lobes and a conserved insertion, a SANT-associated (HSA) domain and an adjacent post-HSA domain at the N-terminus, and AT-hooks and a bromodomain at the C-terminus which bind the acetylated lysins in histone. The ISWI complex contains a central ATPase domain, an autoinhibitory N-terminal (AutoN) domain, a negative regulator of coupling (NegC) domain that flanks the ATPase domain, and a HAND–SANT–SLIDE (HSS) domain at the C-terminus that binds nucleosome and inter-nucleosome DNA. The CHD complex contains a central ATPase domain, arranged in tandem with the chromodomains at the N-terminus that bind the methylated lysins in histone, a NegC domain, and a SANT–SLIDE domain at the C-terminus. The INO80 complex contains a central ATPase domain that includes a large insertion between the RecA-like lobes and an HSA domain at the C-terminus that binds actin-related components. Among the diverse components, ATPase subunits function as motivators that display the DNA/nucleosome-dependent ATPase activity that induces nucleosome assembly and organization, chromatin access, and nucleosome editing (Figure 1B). Each of the remodeler subfamilies contains distinct catalytic ATPases, as well as some associated subunits that collectively generate those who have the potential to form numerous complexes though combinatorial assembly (Table 1) [10]. These large multi-subunit complexes commonly contain specific domains and subunits that are essential for targeting the complex to specific chromatin sites, generally via binding to DNA, modified histones or histone variants. Additionally, these large multi-subunit complexes undergo a high degree of transformation to guarantee the dynamic cellular processes needed to adapt to the changes in the internal and external environments, such as cancer proliferation signals and chemotherapy-induced damage [11]. Therefore, a better understanding of chromatin remodeling is essential for developing new anticancer therapeutic strategies.

### 2.1. SWI/SNF Subfamily in Cancer

**Figure 2:**
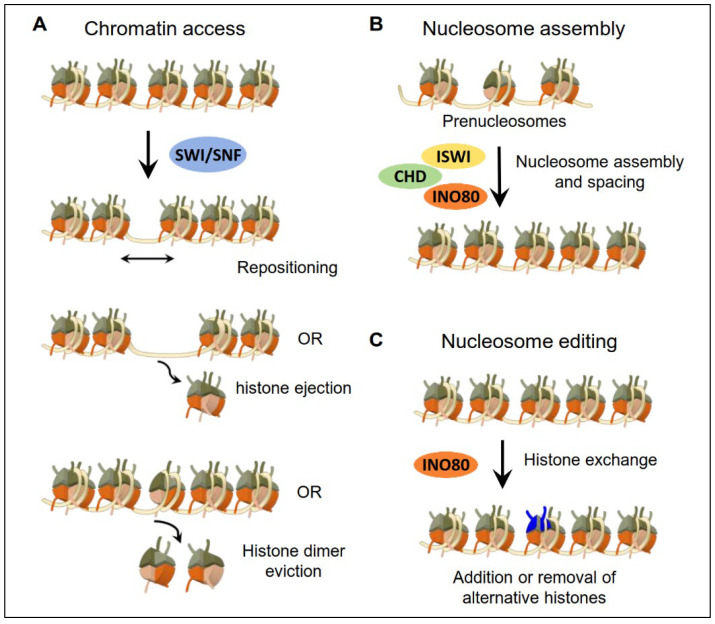
Brief classification of the functions of chromatin remodelers. (**A**) Chromatin access: Primarily SWI/SNF subfamily remodelers restructure chromatin coupling ATP hydrolysis via repositioning nucleosomes, ejecting histone octamers or evicting nucleosomes histone dimers. (**B**) Nucleosome assembly: In particular, ISWI, INO80 and CHD subfamily remodelers re-establish chromatin architecture by the random deposition of histones, the physiological spacing of nucleosomes, and the maturation of nucleosomes. (**C**) Nucleosome editing: INO80 subfamily remodelers alter nucleosome composition via canonical and variant histone exchange, such as histone variants, as marked in blue.

The SWI/SNF complexes, first discovered in yeast, were named for their two targets: the homothallic switching endonuclease, which is a mating-type switch (SWI), and the transforming enzyme sucrose invertase, which is necessary for sucrose non-fermentation (SNF) [29,30]. The SWI/SNF complex is a multi-component complex and usually consists of the conserved DNA-dependent ATPase subunits that act as their catalytic subunit (either Brahma (BRM) or BRM-related Gene 1 (BRG1)), several alternate core subunits (BAF155, BAF170, BAF47, etc.), and a few selected accessory subunits (BAF57, BAF53A/B, BAF60A/B/C and β-actin). SWI/SNF complexes are generally divided into three groups: (1) cBAF complexes containing the BRM/BRG1(SMARCA2/SMARCA4) and associated factors (BAF250a (ARID1A) or BAF250b (ARID1B)); (2) PBAF (polybromo-associated BAF) complexes containing the SMARCA4 ATPase in addition to two subunits (BAF180 (PBRM1) and BAF200 (ARID2)); and (3) non-canonical BAF (ncBAF) complexes (Figure 1B) [31]. Although the biofunction of the SWI/SNF complex is highly dependent on the catalytic activities of the ATPase and helicase domains, the slight difference in attendant subunits gives rise to a diversity of molecular functions of SWI/SNF complexes to adapt to diverse chromosomal functions. The SWI/SNF complex typically promotes chromatin access via repositioning, ejecting nucleosomes and evicting histone dimers to activate or repress gene expression and to facilitate DNA damage repair (Figure 2A); thus, the genetic abnormality of these complexes is closely related to tumor progression and treatment outcomes.

Owing to the rapidly developing tumor genome sequencing technology, mutations in the genes encoding SWI/SNF subunits have been widely detected in multiple tumors, from inactivated biallelic mutations of SMARCB1(BAF47) being present in nearly all rhabdoid tumors to the discovery that ARID1A is mutated in nearly 50% of all ovarian clear cell carcinomas (OCCCs) and ovarian endometrioid carcinomas (OECs) [32,33], and the finding of approximately 40% of clear-cell renal cell carcinoma (ccRCC) cases possess PBRM1 mutations (Table 2) [34]. Collectively, SWI/SNF gene mutations are found in nearly 25% of all cancers. In addition to cancer-related mutations, the aberrant expression of SWI/SNF subunits was also found to be closely linked to tumor initiation and development. Expression of SMARCA4 is silent in 15% up to 50% of human non-small-cell lung cancer (NSCLC) tissues. Additionally, its high mutation rate has been identified in 35% of NSCLC cell lines [35,36]. BRD7, a specific subunit in the PBAF subgroup, is frequently aberrant in breast cancers that possess wild-type instead of mutant p53 [37]. The aberrant expression of SWI/SNF subunits was also found to be closely linked to drug response. For example, SMARCA4 loss shows synthetic lethality with CDK4/6 inhibition in NSCLC [38] and causes a long-lasting major response after pembrolizumab treatment in thoracic malignant rhabdoid-like tumors [39]. Thus, understanding how the SWI/SNF complex contributes to the tumorigenesis process sparks a booming interest in finding SWI/SNF-based therapies for cancer.

### 2.2. ISWI Subfamily

The ISWI gene, which was first discovered in Drosophila, is highly conserved across all species, indicating its essential role in organisms. In humans, there are two ISWI orthologs, hSNF2L (SMARCA1) and hSNF2H (SMARCA5), which have distinct functions. SNF2H is a widely expressed protein and is essential for early embryonic development [86], whereas SNF2L is found at greater levels in terminally differentiated testes, ovaries, and neurons, and delivers tissue-specific effects. In Drosophila, the ISWI subfamily were identified in three different complexes: NURF (nucleosome-remodeling factor), CHRAC (chromatin assembly complex), and ACF (ATP-utilizing chromatin assembly and remodeling factor) (Figure 1B) [87,88]. The ISWI subfamily generally induces the initial prenucleosomes to assemble into canonical octameric nucleosomes, as well as mediating nucleosomes to space themselves at relatively fixed distances (Figure 2B). In humans, both the hSNF2L and hSNF2H ATPase subunits can form stable complexes with all of the accessory subunits that expand the functional ISWI complex members; meanwhile, this takes place with low genetical redundancy. Owing to the functional diversity, the ISWI complex is involved in multiple aspects of cell physiology and pathology, including malignant transformation and progression.

The core ATPase subunit SMARCA1 is ubiquitously expressed in human tissues, but distinct functional roles of SMARCA1, as an oncogenic or tumor suppressor, were observed depending on the tumor types. For example, mutations of SMARCA1 were found to be closely related to NSCLC metastasis. A higher SMARCA1 level was associated with poor overall survival in NSCLC [56]. The oncogenic effects of SMARCA1 were additionally identified in lung and cervical cancer and were found to result in survival and cell cycle progression [89]. On the contrary, SMARCA1 was also identified as a tumor suppressor, probably “for which loss of expression was found in soft tissue sarcoma [19] and silenced in gastric cancer cells due to aberrant methylation [57]”. SMARCA5, the other core ATPase subunit, is frequently overexpressed in various tumors, including in breast cancer [58], gastric cancer [59], acute myeloid leukemia (AML) [60], and pancreatic ductal adenocarcinoma (PDAC) [61]. In addition, the chromosomal translocation t (4; 22) (q31; q12) occurring in the genomic locus of SMARCA5, which generates an EWSR1–SMARCA5 fusion protein via the in-frame fusion of EWSR1 to the last exons of SMARCA5 in extraskeletal Ewing sarcoma/PNET, was identified as having tumorigenic potential [55]. Recent studies found that a circRNA derived from of the SMARCA5 gene (circ-SMARCA5) is involved in the occurrence of several cancers [90], such as circ-SMARCA5 in bladder cancer, which was found to be a potential prognostic marker correlated with advanced tumor features and poor survival [62], and circ-SMARCA5 in breast cancer, which was observed to be silent and correlated with the drug sensitivity of breast cancer cell lines [63]. In addition to these core subunits, noncatalytic subunits are also found to be dysregulated in tumor progression (Table 2).

### 2.3. CHD Complex

Originally discovered in Drosophila, chromodomains were found to promote the formation of heterochromatin [91]. The CHD complex is characterized by two chromodomains arranged in tandem and an ATPase/helicase domain near the N-terminus [92]. The CHD complex comprises at least nine members. Among these, CHD1 and CHD2 are the only ones in this subfamily that possess a DNA-binding domain in their C-terminus that endows CHD1 and CHD2 with direct DNA binding ability [93]. In contrast to CHD1 and CHD2, the subunits CHD3 and CHD4 have a coiled-coil domain in their C-terminus to promote protein–protein interactions, and they can form a complex—namely, the nucleosome-remodeling deacetylase (NuRD) complex—which is the most well-studied member of this subfamily (Figure 1B) [94]. The NuRD complex is a transcriptional repressor that has been extensively investigated due to its important role in cancer progression, particularly in DNA damage response [95,96,97]. The remaining members of this subfamily, CHD6, CHD7, CHD8, and CHD9, are distinguished by tandem Brahma and Kismet (BRK) domains within the C-terminus, which is often associated with chromodomains [98]. In summary, due to the important domains in the CHD family subunits, emerging studies have indicated that the CHD complex is a vital player, encompassing several regions of functional importance.

Multiple studies have shown that genetic and expressional alterations to CHD genes might be strongly correlated with cancer pathogenesis (Table 2). CHD1 has been documented as an essential tumor suppressor and has a strong association with prostate cancer [64]. Mutations and homozygous deletions in CHD1 have been identified in prostate cancers [65], particularly in Chinese patients, where the deletion state was identified in 18% of the cohort [66]. Zhang et al. identified that the loss of CHD1 enables the emergence of antiandrogen resistance in metastatic prostate cancer [67]. Recently, whole-exome sequencing showed that recurrent mutations occurred in the epigenetic modifier molecule CHD2 in 15% of breast implant-associated anaplastic large-cell lymphoma (BI-ALCL) cases [68]. Additionally, Hill et al. conducted a meta-analysis to identify the change in the mutational status from baseline samples to samples of disease progression, and found that CHD2 was one of the present mutations of interest in mantle-cell lymphoma (MCL) [69]. NuRD complex is the most well-studied member of this subfamily, and the disruption of the NuRD function has also been shown to be implicated in oncogenesis. For example, CHD4, an important member of the NuRD complex, has been confirmed to play an essential role in chemoresistance via the sequencing analyses of chemoresistant pediatric AML patients [70].

Another study that analyzed spinal schwannomas and paired blood samples using whole-genome sequencing found that CHD4 was in the gene list and had the highest mutation frequency of cancer-related genes [71]. CHD5 was also discovered to be frequently lost or silenced in high-risk glioma [72] and linked to poor prognosis in neuroblastoma (NB) and several adult cancers [73]. Moreover, genomic aberration was also observed in other CHD genes, such as CHD6 mutation in transitional cell carcinoma (TCC) [74], high mutation rates of CHD7 and CHD8 in CpG islands of methylator phenotype 1 subgroups of colorectal carcinomas (CRCs) [75], and in the recurrent rearrangement of CHD7 in tobacco-smoking small-cell lung cancer patients [76]. Thus, the observation of the genetic alterations harbored in the genes encoding the CHD complex in cancer makes this chromatin-remodeling complex worthy of attention.

### 2.4. The INO80 and SWR1 Family

Similar to most chromatin-remodeling complexes, the INO80 complex is highly evolutionarily conserved, with high homology in the ATPase subunit, and is relatively conserved in the composition of individual complexes [99]. SWR1 is an ATP-dependent chromatin-remodeling complex that is closely related to INO80, which shares several subunits. Generally, the INO80 and SWR1 complex are composed of a heterohexamer of RuvB-like protein that functions as ATPase, nuclear actin and actin-associated proteins, and a few specific accessory subunits (Figure 1B) [100]. For instance, the mammalian INO80 complex is composed of INO80, p400, and Snf2-related CBP activator protein. The ATPase subunits of the INO80 subfamily are characterized by a spacer region that separates the conserved ATPase domain, whose activity was proved to be stimulated by DNA and nucleosomes [101]. INO80 mainly binds to the nucleosome-free regions around the promoter and transcriptional start sites (TSS) and participates in organizing the chromatin architecture through shifting nucleosomes and exchanging histone variants (Figure 2B,C) [102,103]. Due to the crucial role of chromatin organizing activity in DNA processing pathways, many studies have shown that the INO80 subfamily complexes play a vital role in directly regulating DNA replication, repair, and transcription regulation. For example, the INO80 complex acts as an essential coactivator during transcription, controlling the biofunction of the YY1 transcription factors in mammals [104].

In recent years, many studies have identified the genome atlas in the INO80 and SWR1 family locus (Table 2) in cancer patients. The publicly funded project, Cancer Genome Atlas (TCGA), which deposits genomic profiles of cancers, was used to analyze the PDAC-associated genomic alterations and found a high frequency of deletions in the gene encoding INO80C observed in PDAC samples. Consistent with these observations, INO80C deletion was identified to be closely associated with a worse prognosis of patients with *KRAS*^MUT^ PDAC and CRC [78]. Wagener et al. demonstrated that the INO80 complex-associated gene NFRKB is a positional candidate in 11q24.3 through copy-number and whole-exome sequencing analysis of MYC-negative Burkitt-like lymphoma with 11q aberration (mnBLL,11q,) which is a subtype of Burkitt-like lymphoma that is based on a new provisional lymphoma category [79,80]. Germline and paired germline somatic comparative analysis of serrated polyposis syndrome (SPS) with a higher risk for CRC found that INO80 was one of the candidate genes with the germline predisposition to this syndrome [81]. Hepatosplenic T-cell lymphoma (HSTL) is a rare and fatal lymphoma, and INO80 mutation was found to be predominant in HSTL with a high mutation rate (21%) [82]. Furthermore, a high expression of INO80 was frequently found in cancer cell lines and tumor tissues, including in lung cancer, colon cancer, and melanoma [83,84,85]. Although many genetic atlases in the INO80 and SWR1 family locus have been revealed in cancer, it remains a contentious issue to study the underlying regulatory mechanisms.

### 2.5. Mechanisms of ATP-Dependent Chromatin-Remodeling Complexes Dysregulation

Given the evidence of dysregulated ATP-dependent chromatin remodelers in cancer, it is worth considering the mechanisms of the upstream regulator of ATP-dependent chromatin-remodeling complexes first.

The levels of the core ATP-dependent chromatin remodelers vary substantially during development, and from one tissue or cell type to another. Recent work has uncovered several regulatory mechanisms of expression, including gene point and frameshift mutations, deletions, copy number variants (CNVs), and protein activity, as well as expression operating at the transcriptional, post-transcriptional and post- translational levels. For example, the gene encoding the hSNF5 subunit in the SWI/SNF complex was identified as harboring bi-allelic loss-of-function mutations in nearly all early childhood malignant rhabdoid tumors (MRTs). A similar scenario has been identified in MRT cell lines [105]. Furthermore, the activity of ATP-dependent chromatin remodelers is regulated by extracellular and cytoplasmic signals. For instance, the ATPase activity and the associated nucleosome mobilization potential can be aroused by the DNA damage response [106]. Evidence of post-transcriptional regulation was proved by the demonstration that MiR-221 and miR-222 inhibit the SWI/SNF complex subunit ARID1A [107]. Additionally and more importantly, LncRNA CASC15 competes with miR-221 and thereby reverses the repression effect by miR-221 on ARID1A [108]. Similarly, LncRNA DLEU1 influences the expression of SMARCD1 through interaction with miR-490-3p in epithelial ovarian carcinoma [109]. Accumulating evidence also indicates the involvement of post-translational mechanisms in the dysregulation of remodelers in cancer. For instance, the SWI/SNF complex ATPase SMARCA4 is under the control of ubiquitination-mediated degradation, regulated by the SCFFBW7 E3 ubiquitin ligase complex, inducing gastric cancer metastasis repression [110]. In the case of the SWI/SNF complex subunits BAF155 and BAF57, it was observed that BAF57 was stabilized by BAF155, which blocks the binding of E3 ubiquitin ligase TRIP12 to BAF57, thereby inhibiting ubiquitination-mediated degradation [111]. The DNA damage response is a tightly controlled process and is crucial to cancer onset and progression. The INO80 complex undergoes phosphorylation mediated by the Mec1/Tel1 kinases when exposed to DNA-damaging agents and modulates checkpoint responses, thereby activating significant DNA repair processes [112]. Together, this information indicates that the levels of remodeler complex vary during cancer progression and treatment, and subsequent studies will be critical for refining our understanding of the upstream regulatory mechanisms.

## 3. Effects of Chromatin Remodeler Deregulation on Cancer Progression

**Figure 3:**
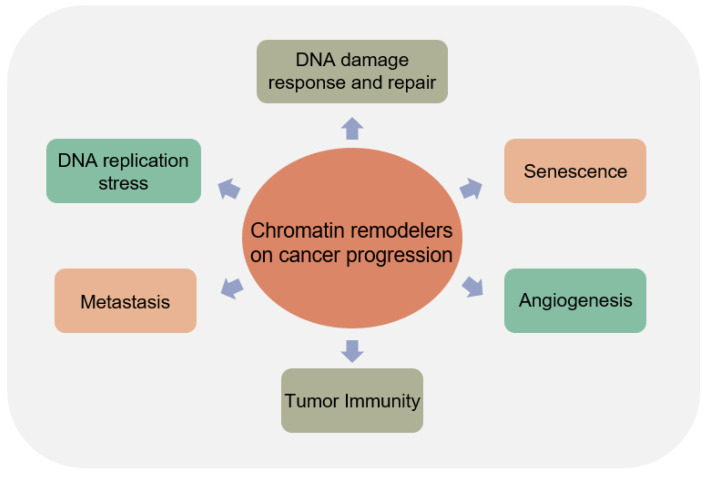
Effects of chromatin remodeler deregulation on cancer progression. Dysregulation of chromatin remodelers impact cancer progression through cellular processes such as DNA damage response and repair, DNA replication stress, senescence, metastasis, angiogenesis, and tumor immunity.

The chromatin-remodeling complex is an important regulator that influences various cell function and pathological processes. The important role of the chromatin-remodeling complex in tumorigenesis and development has gradually emerged. Genomic alterations or activity deregulation in the chromatin-remodeling complex components may alter the progression of tumor cells completely. In general, chromatin-remodeling signaling can impact DNA damage response and repair, DNA replication stress, senescence, metastasis, angiogenesis, and tumor immunity (Figure 3).

### 3.1. DNA Damage Response and Repair

DNA damage induced by chemicals and natural genotoxic agents, such as γ-radiation and UV light in the environment, may lead to gene mutations, whose accumulation is an important process in carcinogenesis. Therefore, upon the onset DNA damage, the DNA damage response (DDR) and repair machinery can sense and activate damage signaling and can then recruit repair factors and trigger cell senescence or programmed cell death, which is essential for DNA damage repair and for impeding the propagation of corrupted genomic information. ATP-dependent chromatin remodelers have been implicated in DDR through a mechanism that depends on increasing nucleosome mobility via ATPase activity. In recent years, a study from our team found that a chromatin remodeler known as MORC2 (microrchidia family CW-type zinc finger 2) played an emerging role in DDR. Upon DNA damage, MORC2 was recruited to the damage site and was PARylated by PARP1, resulting in the activation of the ATPase and chromatin-remodeling activities of MORC2 and the stimulation of the DNA damage response [113]. Notably, we uncovered a new role for acetylated MORC2 in DNA damage-induced checkpoint control [114]. Emerging evidence has shown that chromatin remodelers play a central role in checkpoint control during the damage response. For example, the chromatin remodeler ALC1 (Amplified in Liver Cancer 1, also known as CHD1L) was identified as a key player in catalyzing PARP1-stimulated nucleosome sliding and in controlling checkpoint regulation in response to DNA damage [115]. It was also found that ALC1 deficiency reduced chromatin accessibility as well as the associated repair factors around the damage site, therefore resulting in PARPi sensitivity [116]. ATRX is a chromatin remodeler, and its deficient cells were found to exhibit a defect in DNA repair synthesis and sister chromatid exchange formation at exogenously induced DSBs [117]. The discovery that cancer cells rely on ATRX-mediated DNA repair provides a potential therapeutic strategy to sensitize cancer cells to genotoxic chemotherapy and radiotherapy. Similarly, the SWI/SNF subfamily ATPase SMARCA4 is brought to the broken DNA ends upon DNA damage and is subsequently deacetylated by SIRT1. This stimulates ATPase activity to remodel chromatin and increases the associated homologous recombination (HR) process [118].

### 3.2. DNA Replication Stress

Tumor cells generally show enhanced replicative stress, triggering a specific stress response. Genome instability is commonly associated with tumorigenesis. DNA replication stress, generally triggered by DNA damage or premature mitosis, is now a well-established link with genomic instability, especially during tumorigenesis and progression induced by oncogenes [119,120]. Chromatin remodelers are just beginning to emerge as important regulators in the replication stress response, as nuclear organization dynamics are key determinants of the replication stress response [121]. Transcription–replication (T-R) conflicts, which occur between replication machinery and co-transcriptional R-loops, impede DNA synthesis and thereby induce DNA breaks, which are detrimental to highly proliferated cells [122]. The INO80 complex has been confirmed to promote the resolution of R-loops and thus prevents replication-induced DNA damage in cancer cells [123]. Similarly, Tsai and colleagues found that the core DNA-binding subunit of the BAF complex ARID1A had a profound impact on DNA replication stress management, indicating the potential treatment strategy of targeting ARID1A-deficient cancers [124]. Given the crucial role of the SWI/SNF complex in transcription and DNA replication, Aleix and colleagues testified the effects of the subunit ATPase SMARCA4 on the regulation of R-loop-dependent DNA breaks [125]. Their results showed that depletion of SMARCA4 impaired chromatin-remodeling activity, thereby inhibiting the resolution rate of R-loop-mediated transcription–replication conflicts, resulting in an increased number of R-loop-dependent DNA breaks and genome instability. Given the important role of telomere integrity in genome stability, the alternative lengthening of the telomere (ALT) pathway that promotes telomere elongation is essential for genome stability. The SWI/SNF-related subunit SMARCAL1 was recently demonstrated to be important for ALT telomere maintenance, indicating the potential crucial role of SMARCAL1 in genome stability maintenance [126].

### 3.3. Senescence

Genomic damage, including replicative stress, the hyperactivation of oncogenes (oncogene-induced senescence, OIS), genotoxic drugs, etc., can cause severe damage unless properly managed by cellular stress responses, such as cellular senescence. Senescence can trigger irreversible cell cycle arrest to limit the proliferation of damaged cells that can propagate corrupted genomic information. Senescent cells are characterized by the senescence-associated secretory phenotype (SASP), producing many proinflammatory cytokines and extracellular enzymes such as matrix metalloproteinases [127]. Senescent cells and SASP from senescent cells have been found to be implicated in tumor progression, and cancer therapy-induced senescence has also been proven to drive tumorigenesis and therapy resistance [128]. Chromatin remodelers, which functionally govern chromatin organization and genome accessibility, are prone to be profoundly altered in aging and damaged organisms [129]. For instance, through the analysis of several senescence models, it was found that BAZ1A, an accessory subunit of the chromatin-remodeling complex, was inhibited in senescence cells. Therefore, BAZ1A may act as a crucial modulator in cellular senescence and may represent a potential target in cancer treatment [130]. Furthermore, the deletion or loss-of-function mutation in ISW2 has been implicated in extending the replicative lifespan of yeast, similar to the longevity effect caused by calorie restriction [131]. This important role of the ISWI complex in aging could also represent a valid anticancer strategy. Given that the genomic alteration of SWI/SNF components has been reported to be associated with various types of human cancers, emerging evidence has confirmed the relationship between SWI/SNF complex dysregulation and oncogene-induced senescence. Oncogene-induced senescence generally functions in a potent tumor-suppressive role. In recent years, the SWI/SNF subunit ARID1B was found to be an important regulator of this type of senescence. A study from Luca et al. has shown that the knockdown of the ARID1B could prevent OIS and induce liver tumors though cooperating with oncogene RAS [132]. Soshnikova and colleagues found that the PHF10 subunit in the PBAF complex of SWI/SNF family interacted with MYC and augmented the MYC-induced genes involved in cell cycle motivation. The depletion of PHF10 induced cell cycle arrest and a senescence-like phenotypes [133]. Recently, ARID1A, a paralog of ARID1B, whose deficiency is implicated in the promotion of cancer progression, was identified as being able to induce senescence and the progression of pancreatic intraepithelial neoplasia (PanIN) [134]. Moreover, the loss of function of the SWI/SNF complex via the deletion of specific subunits such as BRD7, SNF5, or PBR1 resulted in a senescence bypass and was relevant to tumorigenesis [37,135,136]. This indicates that a chromatin remodeler could affect senescence in tumor progression, suggesting that prosenescence therapies could be employed by targeting functional chromatin remodeler-inactivated cancers.

### 3.4. Metastasis

Tumor metastases are the greatest lethal factors for cancer patients. Beginning with the invasion of the tissues surrounding the primary tumor, cancer cells then enter into the bloodstream and finally move to progressively colonize distant organs. Recent studies have revealed the complex involvement of chromatin remodeler in the regulation of tumor metastases. Chromatin remodeler dysfunction has been observed during tumor metastases, and there have also been extensive functional and mechanistic studies on chromatin remodelers in recent years. For example, one of two mutually exclusive ATPase subunits from the mammalian SWI/SNF subfamily, SMARCA4, was proven to be a tumor suppressor in the lung cancer. The inactivation of SMARCA4 was found to promote lineage-specific transformation and early metastatic features [42]. Wang and collogues demonstrated that decreased SMARCA4 promotes colorectal cancer metastasis, depending on the Wnt/β-catenin signaling pathway [137]. However, the functional role of SMARCA4 seems to be rather complicated in tumorigenesis, as both tumor-suppressive and oncogenic roles have been revealed during different stages of pancreatic tumorigenesis [138,139]. Therefore, SMARCA4 may exhibit pivotal roles in a cellularly context-dependent manner. For example, SMARCA4 promotes cell migration and invasion through activating the transcription of the oncoprotein transmembrane glycoprotein Mucin 1 (MUC1) and stimulating the TNF-α/IFN-γ pathway in breast cancer [140]. Additionally, SMARCA4 can induce migration and invasion potential of prostate cancer cells [141]. SMARCA2, the other of two mutually exclusive ATPases subunits of the mammalian SWI/SNF subfamily, was also identified as a key mediator in breast cancer metastasis [142].

ARID1A is another essential subunit in SWI/SNF subfamily, whose deficiency promotes cell migration and invasion in lung adenocarcinoma (LUAD) [143] as well as in breast cancer cells [144]. In addition, Shang et al. demonstrated that ARID1A knockdown drives the metastasis of liver cancer cells by weakening SMARCA4-RAD21 interaction [145]. Notably, Sun et al. found that ARID1A has context-dependent tumor-suppressive and oncogenic roles. Briefly, ARID1A is required for tumor initiation in the early stages of hepatocellular carcinoma, while in later stages, such as in established tumors, ARID1A inhibits tumors progression and metastasis [146]. These observations indicate complex roles of ARID1A in human cancer, and directionally opposite effects should be considered when specifying a treatment strategy. Chromatin-remodeling factor ARID2, which belongs to the PBAF complex in the SWI/SNF subfamily, was found to be expressed at a lower level in metastatic hepatocellular carcinoma tissues and to suppress HCC metastasis via the DNMT1–Snail axis, indicating the great therapeutic potential of targeting the DNMT1–Snail axis in ARID2-deficient HCC [147]. The NuRD complex in the CHD subfamily is an important mediator of epithelial–mesenchymal plasticity, which induces tumor metastasis in breast cancer [148]. Consistently, CHD4, an ATPase subunit of the NuRD complex, was also identified in colorectal cancer as a cancer cell motility regulator [149]. In recent years, studies from our lab have found that the chromatin-remodeling protein MORC2 acts as a crucial oncoprotein and promotes breast cancer metastasis. In addition, we identified a gain-of-function mutation of MORC2 that was associated with cancer metastasis and that revealed a post-translational modification of MORC2, namely GlcNAcylation at threonine 556, which enhances breast cancer cell migration and invasion [150,151,152]. These pivotal roles of chromatin remodelers in tumor metastasis may provide insights into therapeutic translation.

### 3.5. Angiogenesis

Angiogenesis, a multi-step process that forms new capillaries via the pre-existing vasculature, is pivotal for the growth and development of solid tumors via supplying oxygen and nutrients to tumor tissues [153]. Chromatin remodeling is an important epigenetic event for regulating angiogenesis in tumor progression and drug resistance, indicating the important potential implications for antiangiogenic agent treatment in chromatin remodeler-controlled tumors. Chromatin remodelers have been known to be involved in hematopoiesis, and the emerging roles in hematopoietic activity in cancer are just beginning to be characterized [154]. Recent studies have demonstrated that Baf200, a subunit of the PBAF complex in the SWI/SNF subfamily, plays a crucial role in malignant hematopoiesis, as the deletion of Baf200 can accelerate tumor progression and shortens the survival of the MLL-AF9-driven leukemogenesis mouse model [155]. Additional evidence shows that the SWI/SNF subfamily is essential for malignant hematopoiesis. Loss of the SWI/SNF subfamily subunit ARID1A was observed in advanced human HCCs and was found to be closely associated with vessel density. Mechanically, ARID1A deficiency causes the epigenetic activation of Ang2; therefore, antiangiogenic therapies against Ang2 in ARID1A-deficient HCC may have good therapeutic effects [156]. Moreover, an analysis of breast cancer xenograft mouse models showed that the SWI/SNF subfamily component of SMARCE1 can protect cells against anoikis and can promote the metastasis of luminal B and basal-like subtypes of breast tumors. This was further underscored by the discovery of the mechanism where SMARCE1 activates the HIF1A/PTK2 pathway, thereby exhibiting an oncogenic role [157]. There is also some evidence that the JARID1B/LSD1/NuRD complex increases cell migration and angiogenesis through the CCL14 chemokine pathway, providing additional knowledge of chromatin remodeler activity in malignant hematopoiesis [158]. As such, there is particular interest in antiangiogenesis for cancer treatment.

### 3.6. Tumor Immunity

In previous studies, it has become clear that our own immune system can be exploited to defend against tumor cells. Immunotherapy has become one of the most prominent cancer treatment strategies in the last decade [63]. In the tumor microenvironment, there are intricate interactions between tumor cells that can, in some cases, manipulate malignant development, and that play an important role in the treatment of tumor patients. Recent evidence has suggested that the epigenetic alterations caused by chromatin mediators, such as chromatin remodelers, cooperatively drive tumor progression and immunotherapy resistance [159]. For instance, the mutations in several of the chromatin remodeler encoding genes, such as ARID1A, ARID1B, and ARID2, have been confirmed to be more likely to benefit from immune checkpoint blockade therapy for NSCLC patients [160,161]. Another recent study has further delineated the tumor-promoting role of the SWI/SNF component SMARCC1 in HCC. SMARCC1 is significantly positively associated with immune infiltration [162]. A CRISPR-Cas9 screen analysis found that ARID2, BRD7, and PBRM1, components of the PBAF complex, sensitized mouse melanoma cells to T-cell cytotoxicity, and PBRM1-deficient murine melanomas were found to be infiltrated by more cytotoxic T cells [163]. Long non-coding RNAs (lncRNAs) represent a subclass of RNAs without coding potential and that are longer than 200 nt. They are widely expressed and play a key role in responding to various cellular functions, ranging from tumor proliferation to tumor-associated inflammation. Chromatin remodelers, in cooperation with lncRNAs, are closely involved in tumor-associated inflammation, such as in the disruption of lncRNA MALAT1, which impairs the recruitment of the chromatin remodeler catalytic subunit SMARCA4 to the promoter regions of IL-6 and CXCL8, resulting in NF-κB pathway activation and HCC progression [164]. The NF-κB pathway is generally considered to be constitutively activated in many cancer types, and to exert protumorigenic functions. For example, the chromatin-remodeling factor SMAR1 was found to transcriptionally upregulate proangiogenic chemokine IL-8, which was dependent on the NF-κB pathway in breast cancer [165]. A recent study illustrated the mediation of the CHD4/NuRD complex on human hepatocellular carcinoma through the regulation of complement gene expression and CD8 T-cell infiltration [166]. From the findings taken together, it can be determined that chromatin remodelers play crucial roles in tumor-associated immunity, and further studies are required to investigate how chromatin remodelers act on the chromosome function-dependent transcriptional act and on the immunomodulation of the tumor microenvironment.

## 4. Targets for Cancer Therapy

### 4.1. Directly Targeted Therapies

Due to the fact that the aberration of the gene-encoding chromatin remodelers are widely observed in a wide array of cancers, drugs that target these genomic aberrations and that are utilized in combination regimens that are able to further enhance anticancer treatment effectiveness provide new insights for the therapeutic strategies in cancer treatment. This has therefore been a focus on the development of the practice of targeting aberrant chromatin remodelers as anticancer agents since the identification of the direct effects on cancer cells in the past two decades (Table 3). Additionally, the chromatin remodeler complex-containing ATPase catalytic subunits make them susceptible to inhibitor strategies via competitive ATP inhibitors and allosteric agents. Other strategies that similarly target catalytic subunits directly by disrupting the protein–protein interaction interfaces, such as stapled peptides and molecules that stabilize or preclude binding to co-regulators, are underway.

Due to the high mutation rate of the SWI/SNF complex in human cancer, numerous studies have focused on developing specific inhibitors to target this complex with precise therapeutic roles. For example, PFI-3, which selectively targets the essential bromodomain of the SMARCA4 and SMARCA2 subunits in BAF complex, has been shown to lower the binding affinity for target gene promoters [168]. Unfortunately, PFI-3 has shown few effects on many cancer types. Additionally, Vangamudi et al. demonstrated that the function of the BAF complex on tumor cells is highly reliant on catalytic ATPase activity, but not on the bromodomain [182]. Therefore, Julien et al. screened the small-molecule inhibitors for ATPase activity of SMARCA2. Fortunately, they found the compound 14, a dual allosteric small-molecule inhibitor that can block both SMARCA2 and SMARCA4 ATPase activity, thus exhibiting striking effects on SMARCA4-deficient lung-cancer models [169]. Further studies have confirmed the antitumor effect by these compounds in uveal melanoma [183]. In addition, Mélin and colleagues recently identified a compound called LM146, which targets the PBAF complex component PBRM1. Further examination of its effects on PBAF-dependent function in tumor development is necessary to fully decipher [173]. Other selective inhibitors that target BRD7/9 have emerged recently, such as the compounds LP99 [174], BI-7273 and BI-956456 [177], GNE-375 [178], and I-BRD9 [179]. BI-7273 and I-BRD9 have been developed and further proven to have an anticancer effect in AML models [179,184]. These highly selective inhibitors that expand the chemical antitumor toolbox will be important for the translational implications of targeting the chromatin-remodeling complex and may be a potent therapeutic strategy in tumors.

However, further progress in targeted cancer therapy has been limited, as not all of the functional genetic alterations are druggable with current conventional approaches. In recent years, targeted protein degradation has been pursued as another targeting strategy because of its great potential to degrade proteins that were previously considered “undruggable”. Briefly, a proteolysis-targeting chimera (PROTAC) molecule harnesses the ubiquitin–proteasome system (UPS), which consists of two ligands joined by a linker that brings the protein of interest to the E3 ubiquitin ligase and induces its ubiquitylation and subsequent degradation, to precisely target the protein for degradation [185,186]. Given the great progress made by academia in recent years, targeted protein degradation offers new options to target the aberrant chromatin remodeler complex in cancers. In recent years, research on compounds that degrade the ATPase and BRD7/9 subunits of SWI/SNF has developed rapidly [170,175,180,187]. For instance, David Remillard created a BRD9-directed degrader (dBRD9) and confirmed the activity of dBRD9 in cellular models of human AML [180]. Then, Zoppi et al. developed a highly selective and rapid degrader, namely VZ185, which dually targets BRD9 and BRD7, indicating that it may be an important tool for the exploration of therapeutic strategies [175]. Due to the concept that SMARCA4-mutant cancers are vulnerable to SMARCA2 inhibition [188], selectively degrading SMARCA2 has been an important therapeutic strategy. A recent study by Farnaby developed an optimized chemical ACBI1 that cooperatively targets SMARCA2, SMARCA4, and PBRM1, exhibiting significant antiproliferative and cell death-inducing effects in SMARCA4-mutant cancer cells [187]. Recently, a new PROTAC degrader targeting SMARCA2 and SMARCA4, called AU-15330, was found to have great preferential cytotoxicity at low concentrations in AR/FOXA1-driven prostate cancer [170]. These findings exemplify the potential of PROTAC-based targeting approaches that enable the selective targeting of previously intractable targets. Additionally, molecular glue compounds are an emerging technique that target protein degradation but that have also undergone extensive development in recent years [189]. These compounds can rapidly degrade previously inaccessible targets, which may act as a viable cancer therapeutic strategy. However, the chromatin-remodeling complexes applied context-dependent roles and exhibited variable expression patterns in several human tumors, such as SMARCA2, which showed context-dependent oncogenic and tumor suppressor roles and lost its expression in SCCOHTs and in a subset of NSCLC. Therefore, a high degree of context-specificity should be taken into consideration when conducting therapeutic approaches using such molecules.

### 4.2. Indirectly Targeted Therapies

**Figure 4:**
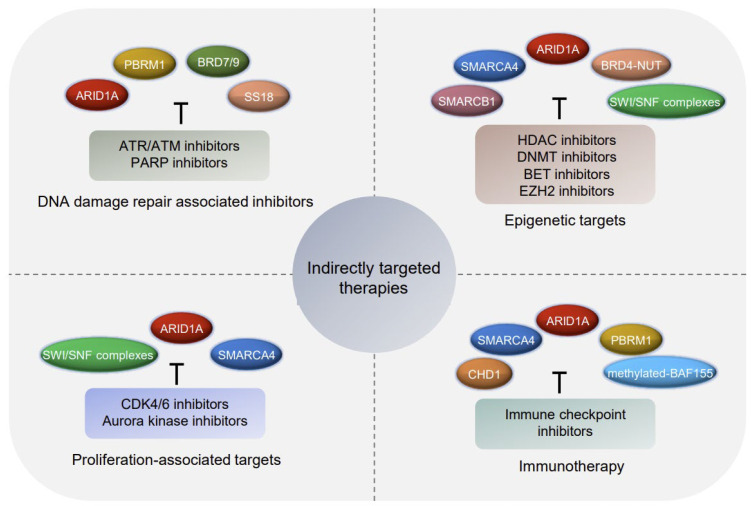
Overview of indirectly targeted therapies that exploit aberrant chromatin remodeling in cancer. Specific vulnerabilities in cancer cells caused by loss-of-function in a chromatin-remodeling situation might be targeted for cancer therapy. Available inhibitors for each target involved in the genetic defect’s vulnerabilities conferred by aberrant chromatin remodeling are listed.

Although the target approach has been extensively developed, since directly restoring the function of the tumor suppressor is currently difficult to achieve, specific vulnerabilities in cancer cells caused by loss-of-function in a tumor suppressor might be targeted for cancer therapy. About 20 years ago, Hartwell and colleagues proposed a genetic concept of synthetic lethality based on genetic information of a disease to drive drug discovery [190]. Since then, applying functional genomic screens to find novel vulnerabilities in cancer cells in line with the defined genetic defects has become a widely used approach to identify novel targets, as well as in the field of combination regimens (Figure 4). For example, ARID1A is identified as frequently harboring loss-of-function mutations across a wide variety of human tumors. Using the functional genomic screen of the vulnerabilities conferred by ARID1A mutation, Helming et al. found that a paralog of ARID1A called ARID1B, which encodes for mutually exclusive BAF subunits, was preferentially required for the proliferation of cancer cell that harboring mutant ARID1A [191]. Mechanistically, the inactivation of ARID1A resulted in the defective control of targeting the BAF complex to a range of genomic regions, but the ARID1B-containing BAF complex still remained intact in the ARID1A-mutant cells, thereby conducting some of the functions to maintain a complex function. Thus, ARID1B is required for chromatin accessibility regulation in ARID1A-deficient colorectal carcinoma cells and OCCCs [192]. Similarly, as we mentioned above, SMARCA4-mutant cancers are vulnerable to SMARCA2 inhibition [188].

#### 4.2.1. DNA Damage Repair Associated Inhibitor

Previous studies have suggested potential roles for chromatin remodelers in DNA repair, such as loss of ARID1A resulting in compromising DNA damage repair. Recent studies have revealed the feasibility of PARP inhibitors in treating patients with ARID1A-defective cancers (Figure 4) [193]. In addition, loss of ARID1A renders cancer cells highly sensitive to combined therapy with PARP inhibitors and ionizing radiation [194]. Moreover, as tumorigenesis in ARID1A-deficiency was also dependent on activation of the PI3K/AKT pathway, the PI3K/AKT inhibitors in combination with PARP inhibitors are a promising strategy for patients with ARID1A-mutant tumors. The inactivation of PBRM1 has been reported to occur frequently in cancers. Roman et al. found that replication stress is greatly elevated in PBRM1-defective cancers cells, and thereby, they treated PARP and ATR inhibitors via a PBRM1-defective ccRCC model. Synthetic lethality effects were observed in this xenograft model [195]. Similarly, a recent study from Hagiwara demonstrated that PARP1 has a good prediction effect as a biomarker to predict PD-L1 blockade response in PBRM1-mutated ccRCC patients, showing good prediction effect [196]. In addition to PBRM1, BRD7 and BRD9 were also identified as potential therapeutic markers to predict synthetic lethality under the treatment with chemotherapeutic drugs and PARP inhibitors [197,198]. Furthermore, the DNA damage repair-associated inhibitors ATR/ATM in combination with ARID1A-deficiency or BAF complex -inhibition functionally synergize, suggesting a potential synthetic lethal strategy to target tumor cells [199,200]. Together, these studies provide important indications of the synergy between chromatin remodeler inhibitors and DNA damage repair-associated inhibitors, especially in combination with chemotherapeutic agents.

#### 4.2.2. Proliferation-Associated Targets

Owing to the essential roles of the chromatin-remodeling complex in the survival and proliferation of cancer cells, the loss of function or dysregulation of several subunits that disrupt the chromatin-remodeling function could lead to genetic vulnerabilities in proliferation-associated signaling pathways. For instance, cyclins and cyclin-dependent kinases (CDKs) are inessential for cell proliferation, and cyclin D-CDK4/6 plays important roles in the uncontrolled cell proliferation of many tumors. The Cyclin D-CDK4/6 pathway was found to be impaired in SMARCA4-defective SCCOHT cells, leading to the therapeutic vulnerabilities to CDK4/6 inhibitors (Figure 4) [201]. Consistent with this effect, SMARCA4-defeciency showed synthetic lethality in CDK4/6-inhibited NSCLCs [38]. Based on the mechanism of the preclinical efficiency of CDK4/6 inhibitor acting on SMARCA4-defective tumors, a phase I study of the CDK4/6 inhibitor Ribociclib (LEE011) was conducted in pediatric patients with rhabdoid tumors harboring SWI/SNF subunits with a high mutation rate [202]. Moreover, the kinase inhibitor ponatinib was found to be synthetically lethal when targeting the SMARCA4-mutant SCCOHT through its inhibition of multiple targets in the receptor tyrosine kinase (RTK) family [203], and the inhibitor dasatinib was also confirmed as an effective agent for ARID1A-defective ovarian clear cell tumors based on the abnormal regulation of the cell cycle [204]. In addition, kinase A has been identified as having a synthetic lethal interaction with ARID1A. ARID1A is required for G2/M transition and mitotic entry, and pharmacological perturbations of AURKA have been found to selectively limit the growth of ARID1A-deficient CRC cells [205]. However, a phase 2 trial of kinase A inhibitor alisertib conducted in pediatric patients showed little antitumor activity when using a single agent [206]. This problem may be improved by adopting genetic context-dependent patient selection for druggable targets, or by using it in combination with other therapeutic agents.

#### 4.2.3. Epigenetic Targets

Given the important role of chromatin remodelers in the epigenetic regulation of human cancers, the combination of chromatin remodeler-associated agents with epigenetic therapy may display clinical benefits for patients. Epigenetic therapies that target aberrant DNA methylation and the post-translational modifications of histones have been well-developed. The recurrent loss-of-function mutations in the genes encoding SMARCA4 have been recently identified to have significant relevance to the sensitivity of HDAC (histone deacetylase), DNMT (DNA methyltransferase), and EZH2 (enhancer of zest homolog 2) inhibitors in SCCOHT patients (Figure 4) [207]. SAHA is a pan-HDAC inhibitor, and it was found to significantly inhibit the progression of ovarian cancer-harboring ARID1A mutations and to significantly prolong the survival of tumor-inoculated mice. This study provided preclinical rationales for pan-HDAC inhibitors in the treatment of ARID1A-mutated tumors [208]. In addition, investigations about the SWI/SNF complexes and polycomb-repressive complexes in cancer have indicated that cancers that harbor SWI/SNF subunits mutations or deletions are sensitive to the inhibition of EZH2. For example, SMARCA4-deficient ovarian small-cell carcinomas and SMARCB1-deficient malignant rhabdoid tumors display sensitivity to EZH2 inhibitors [209]. Additional study found that ATRX alterations, such as in-frame fusion, which occur frequently in neuroblastoma, can promote neuroblastoma development. Neuroblastoma-harboring SWI/SNF complexes undergoing genetic alteration have been found to be sensitive to EZH2 inhibitors [210]. NUT midline carcinoma (NMC) is a rare and aggressive squamous carcinoma subtype that is mainly driven by the BRD4-NUT fusion oncoprotein, and BET (bromodomain extra-terminal) inhibitors were found to have a high efficacy in treating NMC [209]. Therefore, epigenetic-targeting drugs, which have been used in selected patient populations, such as in SWI/SNF-defective cancers, may be the key to broadening its application in cancers.

#### 4.2.4. Immunotherapy

The past decade has seen the emergence of cancer immunotherapies in multiple solid and hematologic malignancies. Knowledge of chromatin remodeling in the regulation of immunotherapies, especially the immune checkpoint inhibitors (ICIs) in cancer, has yielded several promising therapeutic strategies that show great benefits to patients. Genetic alterations in ARID family members have been revealed to be related to sensitivity to ICI therapy in cancer [211], such as in NSCLC [160], EBV-positive gastric cancers [212], and ovarian cancer (Figure 4) [213,214]. For example, ARID1A alters sensitize tumors to immune checkpoint blockade, suggesting a potential candidate for immunotherapy [215]. Studies by Shen and colleagues have revealed that treatments with the anti-PD-L1 antibody on ARID1A-deficient ovarian tumors demonstrate promising therapeutic activity in preclinical models [213]. Another study by Fukumoto observed that combined treatments with the HDAC6 inhibitor and anti-PD-L1 in ARID1A-mutated cancers showed efficient antitumor effects caused by improved cytotoxic T-cell activity [216]. In addition, SMARCA4-mutant tumors were more sensitive to ICIs, and treatment with ICIs was associated with improved outcomes. SMARCA4 alterations in NSCLC were found to boost higher response rates to anti-PD-L1 treatment [217]. Furthermore, a novel small molecule, namely IACS-010759, which is an inhibitor of OXPHOS undergoing clinical development, showed a potent antitumor efficacy in SMARCA4-mutant lung cancers [218]. Bai et al. identified mutant-SMARCA4 as a predictive biomarker of ICB efficacy in EBV-associated gastric cancer [219]. Additionally, methylated-BAF155 [220], PBRM1 [221,222], and CHD1 [223] have been reported to be closely related to immunotherapy responses and may act as potential targets in clinical applications. Thus, studies focusing on the mechanisms and applications of chromatin-remodeling alterations in cancer immunotherapy should provide mechanism references and broaden the application of immune checkpoint blockade to patients with the selected subtypes based on the genomic defects of chromatin remodeling.

## 5. Outlook

In this review, we have summarized the composition of ATP-dependent chromatin-remodeling complexes and have provided a detailed description of the genomic alterations to the subunit complexes, as well as their associated dysfunction in cancer development. This will provide new insights into cancer progression and will offer novel therapeutic strategies for chromatin remodeling in defective cancers. Notably, with the emerging technology for screening for new small inhibitors and immune therapy, combination treatments targeting malfunctioning molecules or pathways based on patient selection strategies may achieve more effective and fewer side effects over conventional chemotherapy.

ATP-dependent chromatin remodelers are large chromatin-remodeling machines that are connected to chromatin and nucleosome function, and thus contribute greatly to many chromosomal associated functions and various aspects of the cellular process. Thanks to the rapid development of large-scale sequencing technology, a new class of the somatic and inherited genomic variants that encode the chromatin remodelers have been identified as risk factors of cancer. Various altered chromatin-remodeling subunits in tumors have been confirmed to contribute to cancer phenotype, such as resistance to DNA damage repair, DNA replication stress, senescence, angiogenesis, metastasis and tumor immunity, etc. Additionally, the high flexibility of the composition and conformation of the remodeling complex in cancer could lead to interactome dynamics that affect the chromatin-remodeling function in a context-dependent way. Therefore, it will be meaningful to determine the oncogenic genomic alterations and delineate their biological functions in a context-dependent way, and to thus uncover additional novel drug targets.

The concept of therapies that are based on synthetic effects have broadened the application of currently available drugs with monotherapy or combination therapies. Thus, revealing the interplay of chromatin remodeling associated with oncogenic pathway signaling and determining the genomic alterations that cause tumor vulnerabilities may provide new guidelines for synergistic drug combination strategies for cancer therapy. New small-molecule drugs such as PROTAC molecules and molecular glues that induce the degradation of targets that are intractable by conventional pharmacological methods are emerging strategies for targeted therapy in cancer. Furthermore, as the improvements in sequencing technologies such as single-cell sequencing enable us to detect genomic features from single cells, data with high resolution and richness can be acquired to uncover the new roles of the chromatin-remodeling complex in tumor initiation and development. With predictive biomarkers for patient selection, precision medicine based on chromatin-remodeling dysfunction will provide new therapeutic avenues for cancer therapy.

## Figures and Tables

**Table 1:** Components of chromatin-remodeling complexes.

Remodeling Complex	Gene Symbol	Protein Name in Complex	Brief Descriptions of Their Roles
SWI/SNF Family			
**cBAF complex**	SMARCA4	BRG1	SMARCA4, as catalytic subunit binds with and pumps DNA along the nucleosome [12].
	SMARCA2	BRM	SMARCA2 is a SMARCA4 homolog and processes helicase and ATPase activities, which is a role highly similar to SMARCA4 [12].
	ACTL6A/B	BAF53A/B	ACTL6A/B can form a heterodimer with ACTB, and bridge the ATPase and base complex [12].
	SMARCJ1/2/3	BCL7A/B/C	SMARCJ1/2/3 share strong sequence similarity, and bind with SMARCA4 [13].
	SMARCD1/2/3	BAF60A/B/C	SMARCD1/2/3 facilitate base complex organization [12].
	SMARCB1	BAF47	SMARCB1 mediates interaction of the complex with the nucleosome [12].
	SMARCE1	BAF57	SMARCE1 facilitates base complex organization [12].
	SMARCC1/2	BAF155/170	SMARCC1/2 serve as scaffold in the base module organization [12].
	ACTB	β-actin	ACTB forms a heterodimer with ACTL6A and bridges the ATPase and base complexes [12].
	SMARCL1	SS18	SMARCL1 associates with SMARCA2 and SMARCA4 [14].
	DPF1/3/2	BAF45B/C/D	DPF1/3/2 are quantitatively associated with SMARCA4 [15].
	ARID1A/B	BAF250A/B	ARID1A/B serve as a structural core in the base complex organization [12].
	SMARCM1/2	BCL11A/B	SMARCM1/2 bound to the cBAF complex with great stabilities [13].
**PBAF complex**	SMARCA4	BRG1	SMARCA4 as catalytic subunit binds with and pumps DNA along the nucleosome [12].
	SMARCA2	BRM	SMARCA2 is a SMARCA4 homolog, and processes helicase and ATPase activities which is highly similar to SMARCA4 [12].
	ACTL6A/B	BAF53A/B	ACTL6A/B can form a heterodimer with ACTB, and bridge the ATPase and base complex [12].
	SMARCJ1/2/3	BCL7A/B/C	SMARCJ1/2/3 share strong sequence similarity, and bind with SMARCA4 [13].
	SMARCD1/2/3	BAF60A/B/C	SMARCD1/2/3 facilitate base complex organization [12].
	SMARCB1	BAF47	SMARCB1 mediates interaction of the complex with the nucleosome [12].
	SMARCE1	BAF57	SMARCE1 facilitate base complex organization [12].
	SMARCC1/2	BAF155/170	SMARCC1/2 serve as scaffold in the base module organization [12].
	SMARCL1	SS18	SMARCL1 associates with SMARCA2 and SMARCA4 [14].
	ACTB	β-actin	ACTB forms a heterodimer with ACTL6A, and bridges the ATPase and base complex [12].
	SMARCG4	PHF10	SMARCG4 can readily access the H3 tails [16].
	ARID2	BAF200	ARID2 acts as the structural core for assembly of the DNA-binding lobe [16].
	SMARCI1	BRD7	SMARCI1 plays a role in H3 recognition [16].
	PBRM1	BAF180	PBRM1 provides a structural basis for histone tail binding [16].
**ncBAF complex**	SMARCA4	BRG1	SMARCA4, as a catalytic subunit, binds with and pumps DNA along the nucleosome [12].
	SMARCA2	BRM	SMARCA2 is a SMARCA4 homolog, and processes helicase and ATPase activities, which is highly similar to the role of SMARCA4 [12].
	SMARCJ1/2/3	BCL7A/B/C	SMARCJ1/2/3 share strong sequence similarity, and bind with SMARCA4 [13].
	SMARCD1/2/3	BAF60A/B/C	SMARCD1/2/3 facilitate base complex organization [12].
	SMARCB1	BAF47	SMARCB1 mediates interaction of the complex with the nucleosome [12].
	SMARCE1	BAF57	SMARCE1 facilitate base complex organization [12].
	SMARCC1/2	BAF155/170	SMARCC1/2 serve as a scaffold in the base module organization [12].
	ACTL6A/B	BAF53A/B	ACTL6A/B can form a heterodimer with ACTB and bridge the ATPase and base complex [12].
	SMARCL1	SS18	SMARCL1 associates with SMARCA2 and SMARCA4 [14].
	SMARCI2	BRD9	SMARCI2 contains a bromodomain and a DUF3512 domain, which are essential for the assembly of the ncBAF complex [17].
	BICRA/AL	GLTSCR1/1L	BICRA/AL contribute to the function of chromatin targeting and nucleosome-remodeling [18].
	ACTB	β-actin	ACTB forms a heterodimer with ACTL6A and bridges the ATPase and base complex [12].
**ISWI Family**			
**NURF complex**	SMARCA1	SNF2L	SMARCA1 is an ATPase which engages nucleosomes and is involved in nucleosome substrate binding [19].
	RBBP4	RBAP48	RBBP4 is a WD40 repeat containing histone binding protein and is a component of the NURF complex [20].
	RBBP7	RBAP46	RBBP7 shares high sequence identity with RBAP48, and has high affinity for histones [21].
	BPTF	BPTF	BPTF is Bromodomain and PHD finger containing transcription factor, and a core subunit of the NURF complex [22].
**CHRAC complex**	SMARCA1	SNF2L	SMARCA1 is an ATPase which engages nucleosomes and is involved nucleosome substrate binding [19].
	BAZ1A	ACF1	BAZ1A is ATP-utilizing chromatin assembly and remodeling factor and catalyzes the ATP-dependent assembly of nucleosome arrays [23].
	CHRAC1/2	CHRAC-15/17	CHRAC1/2 are histone-fold proteins, and facilitate ATP-dependent nucleosome sliding [24].
**ACF complex**	SMARCA1	SNF2L	SMARCA1 is an ATPase which engages nucleosomes and is involved in nucleosome substrate binding [19].
	BAZ1A	ACF1	BAZ1A is an ATP-utilizing chromatin assembly and remodeling factor and catalyzes the ATP-dependent assembly of nucleosome arrays [23].
**CHD/mi-2 Family**			
**NuRD complex**	CHD3/4	Mi-2a/b	CHD3/4 are ATP-dependent remodeling enzymes and catalyze the ATP-dependent assembly of nucleosome arrays [21].
	RBBP7	RBAP46	RBBP7 ensures a stable platform for binding histones and involves itself in de novo histone H4 acetylation [21].
	RBBP4	BAP48	RBBP4 is an essential chaperone for histone tetramer deposition on newly replicated DNA [21].
	GATAD2A/B	p66α/β	GATAD2A/B interact and colocalize with MBD2/3 [21].
	HDAC1/2	HDAC1/2	HDAC1/2 participates in the remodeling of chromatin by deacetylating histones [21].
	MTA1/2/3	MTA1/2/3	MTA1/2/3 read histone tails and promoters [21].
	MBD2/3	MBD2/3	DNA-binding and the connexion to methylation PMID: 25796366
**INO80 Family**			
**INO80 complex**	INO80	INO80	INO80 is an ATP-dependent enzyme for chromatin remodeling [25].
	ACTL6A	ARP4	ACTL6A is an actin-related protein, and can hydrolyze or bind ATP [25].
	ACTR5	ARP5	ACTR5 is an actin-related protein, and interacts with the insertion of the Ino80p ATPase domain [25].
	ACTR8	ARP8	ACTR8 is an actin-related protein, and binds core histones [25].
	UCHL5	INO80R	UCHL5 is the deubiquitylating enzyme for histones or other chromatin proteins [25].
	TFPT	INO80F	TFPT is a INO80 chromatin-remodeling complex subunit and recruits the complex to regulatory elements of target genes [26].
	RUVBL1/2	INO80H/J	RUVBL1/2 have ATPase activity and possess DNA/RNA-binding domain [27].
	YY1	INO80S	YY1 recruits the INO80 complex to its DNA-binding sites [25].
	INO80B/C/D	INO80B/C/D	INO80B/C/D involve in DNA recombination and DNA repair [25].
	CCDC95	INO80E	INO80E is a INO80 chromatin-remodeling complex subunit and has a coiled-coil domain [26].
	MCRS1	INO80Q	MCRS1 is a critical histone acetylation regulator with an FHA domain [28].
	NFRKB	INO80G	NFRKB as nuclear factors related to κB bind specifically to NF-κB DNA-binding sites [25].

**Table 2:** Genomic alteration of ATP-dependent chromatin-remodeling factors in cancer.

Tumors	Genomic Alteration	Molecular Functions	Refs.
Ovarian carcinomas	ARID1A mutations	ARID1A mutations induce early transformation of endometriosis into cancer.	[32]
Ovarian clear cell carcinoma	ARID1A mutations	Aberrant ARID1A contributes to the pathogenesis of OCCC.	[33]
Clear cell carcinoma	PBRM1/BAF180 truncating mutations	Truncating mutations of PBRM1 contribute to aberrant chromatin biology.	[34]
Lung cancer	Concomitant loss of BRG1/BRM	Loss of BRG1/BRM is correlated with poor prognosis.	[35]
Rhabdoid tumors	SMARCB1 biallelic mutations	Inactivation of SMARCB1 upregulates cell cycle progression.	[40]
Lung primary tumor	SMARCA4 inactivating mutations	Inactivation of SMARCA4 contributes to the development of lung primary tumor.	[41]
Breast tumor	Low BRD7 expression	Low BRD7 expression promotes tumorigenicity.	[37]
Non-small-cell lung cancer	SMARCA4 loss	SMARCA4 loss exhibits a synthetic lethality with CDK4/6 inhibition.	[38]
Malignant rhabdoid tumor	SMARCA4 inactivation	SMARCA4-deficient causes long-lasting response to pembrolizumab treatment.	[39]
Lung adenocarcinoma	SMARCA4/BRG1 Inactivation	Inactivation of SMARCA4 promotes transformation and early metastasis.	[42]
Leukemia	SMARCD2/ BAF60b loss-of-function mutations	Loss-of-function mutations of SMARCD2 promote acute myeloid leukemia.	[43]
Glioblastoma	SMARCB1/BAF47	A SMARCB1 mutation predisposes to earlier development of glioblastoma.	[44]
Thyroid Tumor	SWI/SNF complex mutations	SWI/SNF complex mutations promote thyroid tumor progression and resistance to redifferentiation therapies.	[45]
Lung cancer	Inactivation of SMARCA2	SMARCA2 promoter hypermethylation plays an oncogenetic role.	[46]
Clear cell meningioma	SMARCE1/BAF57 mutations	SMARCE1 mutations cause spinal and cranial clear cell meningioma. Germline SMARCE1 mutations were found in familial pediatric clear cell meningioma.	[47,48]
Squamous Cell Carcinoma	ACTL6A/BAF53A co-amplified with p63	ACTL6A is co-amplified with p63 and acts as an oncogenic driver in squamous cell carcinoma.	[49]
Synovial sarcomas	SS18: SSX fusion	SS18: SSX fusion acts as an oncogenic driver in synovial sarcomas.	[50]
Endometroid and ovarian clear cell cancers	ARID1A/BAF250A loss-of-function mutations	ARID1A mutations impacts numerous signals important in oncogenesis.	[51]
Colorectal cancer	ARID1B/ BAF250B inactivation mutation	ARID1B inactivation mutation may play a role in microsatellite unstable colorectal cancer.	[52]
Lung cancer	ARID2/BAF200 mutations	ARID2 deficiency increases tumor progression and chemotherapy resistance in lung cancer.	[53]
Lung cancer	PBRM1/BAF180 mutation	PBRM1 mutation may be a negative predictive biomarker for immunotherapy in NSCLC.	[54]
Ewing sarcoma/primitive neuroectodermal tumor	EWSR1-SMARCA5/SNF2H fusion	EWSR1-hSNF2H may act as an oncogenic chromatin-remodeling factor.	[55]
Lung adenocarcinoma	SMARCA1/ SNF2L mutations	SMARCA1 mutations were associated with metastasis.	[56]
Soft-tissue sarcoma	SMARCA1	SMARCA1 loss affects the differentiation process	[19]
Gastric cancer	SMARCA1 aberrant methylation	SMARCA1 loss promotes cancer cell growth	[57]
Breast cancer, gastric cancer, acute myeloid leukemia, pancreatic ductal adenocarcinoma	SMARCA5 overexpressed	Overexpression of SMARCA5 promotes cancer progression.	[58,59,60,61]
Bladder cancer, breast cancer,	circ-SMARCA5 silent	circ-SMARCA5 acts as a potential prognostic marker.	[62,63]
Prostate cancer	CHD1 deletion	CHD1 shows a key role in prostate cancer biology,	[64,65,66]
Metastatic prostate cancer	CHD1 loss	CHD1 loss is a cause of antiandrogen resistance.	[67]
Prostate cancer	CHD1 deletions	CHD1 deletions were correlated with disease phenotype and progression.	[66]
Breast implant-associated anaplastic large-cell lymphoma, mantle-cell lymphoma	CHD2 mutation	CHD2 mutation is an oncogenic event.	[68,69]
Acute myeloid leukemia	CHD4 mutation	CHD4 mutations enrich in primary chemoresistance patients.	[70]
Spinal schwannoma	CHD4 mutation	CHD4 is a frequently mutated cancer-related gene in spinal schwannoma.	[71]
Human cancer	CHD5 deletion	CHD5 deletion controls proliferation, apoptosis, and senescence.	[72]
Neuroblastoma	CHD5 and ARID1A deletion	CHD5 and ARID1A deletion links to poor prognosis of neuroblastoma.	[73]
Bladder cancer	CHD6 aberration	CHD6 aberration might be a hallmark of bladder cancer.	[74]
Colorectal carcinomas	CHD7 and CHD8 mutation	Mutations in CHD7 and CHD8 occurred frequently in CpG island methylator phenotype 1 colorectal carcinomas.	[75]
Lung cancer	CHD7 rearrangement	Recurrently rearrangement of CHD7 occurs in tobacco-smoking small-cell lung cancer patients.	[76]
Human cancers	CHD7 gained/amplified and mutated	CHD7 is associated with poor prognosis in human cancer.	[77]
Pancreatic cancer	INO80C deletion	INO80C deletion is associated with worse prognosis of patients.	[78]
Burkitt-like lymphoma	NFRKB aberration	NFRKB aberration is a positional candidate.	[79,80]
Colorectal cancer	INO80 variants	INO80 is candidate gene with a higher risk for colorectal cancer.	[81]
Hepatosplenic T-cell lymphoma	INO80 and ARID1B mutation	INO80 and ARID1B mutations linked to Hepatosplenic T-cell lymphoma pathogenesis.	[82]
Colon cancer	INO80 haploinsufficiency	INO80 haploinsufficiency suppresses colon cancer tumorigenesis.	[83]
Non-small-cell lung cancer	INO80 highly expressed	INO80 promotes oncogenic transcription and NSCLC tumorigenesis	[84]
Melanoma	INO80 elevated	Elevated INO80 induces melanoma progression.	[85]

**Table 3:** Targeted therapies that directly target the aberrant chromatin remodelers as anticancer agents.

Directly Targets	Associated Cancers	Directly Targeted Agents
SMARCA4 and SMARCA2	Glioblastoma	PFI-3 targets the essential bromodomain and blocks SWI/SNF’s chromatin binding [167,168].
SMARCA2 and SMARCA4	SMARCA4-deficient lung-cancer; uveal melanoma	A dual allosteric small-molecule inhibitor targets ATPase activity of SMARCA2 and SMARCA4 [169].
SMARCA2 and SMARCA4	AR/FOXA1-driven prostate cancer	AU-15330 is a proteolysis-targeting chimera degrader of the SMARCA2 and SMARCA4 [170].
SMARCA2 and SMARCA4	SMARCA4-mutant lung cancer	SMASh degron-mediated SMARCA2 depletion [171].
SMARCA2, SMARCA4, and PBRM1	Unknown	GNE-064 is a chemical probe targeting the bromodomains SMARCA2, SMARCA4, and PBRM1 [172].
PBAF complex	Unknown	LM146 targets the PBAF complex component by blocking the specific bromodomains within the complex [173].
BRD7/9	Unknown	Compounds LP99 is selective inhibitor of the BRD7 and BRD9 bromodomains [174].
BRD7/9	Unknown	VZ185 is a selective and rapid degrader of BRD9 and of its close homolog BRD7 [175].
BRD7/9	Unknown	GSK6776 as a soluble and selective BRD7/9 inhibitor [176].
BRD9	AML xenograft model	BI-7273 and BI-956456 are potent and selective BRD9 bromodomain inhibitors [177].
BRD9	Unknown	GNE-375 is a small-molecule inhibitor of the BRD9 bromodomain [178].
BRD9	Human AML	I-BRD9 is BRD9 bromodomain inhibitor [179].
BRD9	Human AML	Compound dBRD9 bridges the BRD9 bromodomain and the E3 ubiquitin ligase complex for degradation [180].
BPTF	Breast cancer cells	Compound BZ1 targets the BPTF bromodomain [181].
BPTF	Lung cancer cells	Compounds Cpd8 and Cpd10 are highly potent and selective inhibitors of the BPTF bromodomain [22].

## Data Availability

Not applicable.

## References

[1] Michael A.K., Thoma N.H. (2021). Reading the chromatinized genome. Cell.

[2] Moore-Morris T., van Vliet P.P., Andelfinger G., Puceat M. (2018). Role of Epigenetics in Cardiac Development and Congenital Diseases. Physiol. Rev..

[3] Clapier C.R., Iwasa J., Cairns B.R., Peterson C.L. (2017). Mechanisms of action and regulation of ATP-dependent chromatin-remodelling complexes. Nat. Rev. Mol. Cell Biol..

[4] Cheng M.L., Solit D.B. (2018). Opportunities and Challenges in Genomic Sequencing for Precision Cancer Care. Ann. Intern. Med..

[5] Janssen A., Breuer G.A., Brinkman E.K., van der Meulen A.I., Borden S.V., van Steensel B., Bindra R.S., LaRocque J.R., Karpen G.H. (2016). A single double-strand break system reveals repair dynamics and mechanisms in heterochromatin and euchromatin. Genes Dev..

[6] Bell O., Tiwari V.K., Thoma N.H., Schubeler D. (2011). Determinants and dynamics of genome accessibility. Nat. Rev. Genet..

[7] Dai Q., Ye Y. (2022). Development and Validation of a Novel Histone Acetylation-Related Gene Signature for Predicting the Prognosis of Ovarian Cancer. Front. Cell Dev. Biol..

[8] Jenuwein T., Allis C.D. (2001). Translating the histone code. Science.

[9] Maeda M., Takeshima H., Iida N., Hattori N., Yamashita S., Moro H., Yasukawa Y., Nishiyama K., Hashimoto T., Sekine S. (2020). Cancer cell niche factors secreted from cancer-associated fibroblast by loss of H3K27me3. Gut.

[10] De la Serna I.L., Ohkawa Y., Imbalzano A.N. (2006). Chromatin remodelling in mammalian differentiation: Lessons from ATP-dependent remodellers. Nat. Rev. Genet..

[11] Narlikar G.J., Sundaramoorthy R., Owen-Hughes T. (2013). Mechanisms and functions of ATP-dependent chromatin-remodeling enzymes. Cell.

[12] He S., Wu Z., Tian Y., Yu Z., Yu J., Wang X., Li J., Liu B., Xu Y. (2020). Structure of nucleosome-bound human BAF complex. Science.

[13] Kadoch C., Hargreaves D.C., Hodges C., Elias L., Ho L., Ranish J., Crabtree G.R. (2013). Proteomic and bioinformatic analysis of mammalian SWI/SNF complexes identifies extensive roles in human malignancy. Nat. Genet..

[14] Thaete C., Brett D., Monaghan P., Whitehouse S., Rennie G., Rayner E., Cooper C.S., Goodwin G. (1999). Functional domains of the SYT and SYT-SSX synovial sarcoma translocation proteins and co-localization with the SNF protein BRM in the nucleus. Hum. Mol. Genet..

[15] Chugunov A.O., Potapova N.A., Klimenko N.S., Tatarskiy V.V., Georgieva S.G., Soshnikova N.V. (2021). Conserved Structure and Evolution of DPF Domain of PHF10-The Specific Subunit of PBAF Chromatin Remodeling Complex. Int. J. Mol. Sci..

[16] Yuan J., Chen K., Zhang W., Chen Z. (2022). Structure of human chromatin-remodelling PBAF complex bound to a nucleosome. Nature.

[17] Wang X., Wang S., Troisi E.C., Howard T.P., Haswell J.R., Wolf B.K., Hawk W.H., Ramos P., Oberlick E.M., Tzvetkov E.P. (2019). BRD9 defines a SWI/SNF sub-complex and constitutes a specific vulnerability in malignant rhabdoid tumors. Nat. Commun..

[18] Alpsoy A., Dykhuizen E.C. (2018). Glioma tumor suppressor candidate region gene 1 (GLTSCR1) and its paralog GLTSCR1-like form SWI/SNF chromatin remodeling subcomplexes. J. Biol. Chem..

[19] Patil P.A., Lombardo K., Sturtevant A., Mangray S., Yakirevich E. (2018). Loss of Expression of a Novel Chromatin Remodeler SMARCA1 in Soft Tissue Sarcoma. J. Cytol. Histol..

[20] Hart P., Hommen P., Noisier A., Krzyzanowski A., Schuler D., Porfetye A.T., Akbarzadeh M., Vetter I.R., Adihou H., Waldmann H. (2021). Structure Based Design of Bicyclic Peptide Inhibitors of RbAp48. Angew. Chem. Int. Ed. Engl..

[21] Torchy M.P., Hamiche A., Klaholz B.P. (2015). Structure and function insights into the NuRD chromatin remodeling complex. Cell Mol. Life Sci..

[22] Xiong L., Mao X., Guo Y., Zhou Y., Chen M., Chen P., Yang S., Li L. (2021). Discovery of selective BPTF bromodomain inhibitors by screening and structure-based optimization. Biochem. Biophys. Res. Commun..

[23] Fyodorov D.V., Blower M.D., Karpen G.H., Kadonaga J.T. (2004). Acf1 confers unique activities to ACF/CHRAC and promotes the formation rather than disruption of chromatin in vivo. Genes Dev..

[24] Kukimoto I., Elderkin S., Grimaldi M., Oelgeschlager T., Varga-Weisz P.D. (2004). The histone-fold protein complex CHRAC-15/17 enhances nucleosome sliding and assembly mediated by ACF. Mol. Cell.

[25] Conaway R.C., Conaway J.W. (2009). The INO80 chromatin remodeling complex in transcription, replication and repair. Trends Biochem. Sci..

[26] Cox E., Hwang W., Uzoma I., Hu J., Guzzo C.M., Jeong J., Matunis M.J., Qian J., Zhu H., Blackshaw S. (2017). Global Analysis of SUMO-Binding Proteins Identifies SUMOylation as a Key Regulator of the INO80 Chromatin Remodeling Complex. Mol. Cell Proteom..

[27] Matias P.M., Gorynia S., Donner P., Carrondo M.A. (2006). Crystal structure of the human AAA+ protein RuvBL1. J. Biol. Chem..

[28] Garrido A., Kim E., Teijeiro A., Sanchez Sanchez P., Gallo R., Nair A., Matamala Montoya M., Perna C., Vicent G.P., Munoz J. (2022). Histone acetylation of bile acid transporter genes plays a critical role in cirrhosis. J. Hepatol..

[29] Farrants A.K. (2008). Chromatin remodelling and actin organisation. FEBS Lett..

[30] Peterson C.L., Dingwall A., Scott M.P. (1994). Five SWI/SNF gene products are components of a large multisubunit complex required for transcriptional enhancement. Proc. Natl. Acad. Sci. USA.

[31] Mashtalir N., D’Avino A.R., Michel B.C., Luo J., Pan J., Otto J.E., Zullow H.J., McKenzie Z.M., Kubiak R.L., St Pierre R. (2018). Modular Organization and Assembly of SWI/SNF Family Chromatin Remodeling Complexes. Cell.

[32] Wiegand K.C., Shah S.P., Al-Agha O.M., Zhao Y., Tse K., Zeng T., Senz J., McConechy M.K., Anglesio M.S., Kalloger S.E. (2010). ARID1A mutations in endometriosis-associated ovarian carcinomas. N. Engl. J. Med..

[33] Jones S., Wang T.L., Shih Ie M., Mao T.L., Nakayama K., Roden R., Glas R., Slamon D., Diaz L.A., Vogelstein B. (2010). Frequent mutations of chromatin remodeling gene ARID1A in ovarian clear cell carcinoma. Science.

[34] Varela I., Tarpey P., Raine K., Huang D., Ong C.K., Stephens P., Davies H., Jones D., Lin M.L., Teague J. (2011). Exome sequencing identifies frequent mutation of the SWI/SNF complex gene PBRM1 in renal carcinoma. Nature.

[35] Reisman D.N., Sciarrotta J., Wang W., Funkhouser W.K., Weissman B.E. (2003). Loss of BRG1/BRM in human lung cancer cell lines and primary lung cancers: Correlation with poor prognosis. Cancer Res..

[36] Medina P.P., Romero O.A., Kohno T., Montuenga L.M., Pio R., Yokota J., Sanchez-Cespedes M. (2008). Frequent BRG1/SMARCA4-inactivating mutations in human lung cancer cell lines. Hum. Mutat.

[37] Drost J., Mantovani F., Tocco F., Elkon R., Comel A., Holstege H., Kerkhoven R., Jonkers J., Voorhoeve P.M., Agami R. (2010). BRD7 is a candidate tumour suppressor gene required for p53 function. Nat. Cell Biol..

[38] Xue Y., Meehan B., Fu Z., Wang X.Q.D., Fiset P.O., Rieker R., Levins C., Kong T., Zhu X., Morin G. (2019). SMARCA4 loss is synthetic lethal with CDK4/6 inhibition in non-small cell lung cancer. Nat. Commun..

[39] Henon C., Blay J.Y., Massard C., Mir O., Bahleda R., Dumont S., Postel-Vinay S., Adam J., Soria J.C., Le Cesne A. (2019). Long lasting major response to pembrolizumab in a thoracic malignant rhabdoid-like SMARCA4-deficient tumor. Ann. Oncol..

[40] Isakoff M.S., Sansam C.G., Tamayo P., Subramanian A., Evans J.A., Fillmore C.M., Wang X., Biegel J.A., Pomeroy S.L., Mesirov J.P. (2005). Inactivation of the Snf5 tumor suppressor stimulates cell cycle progression and cooperates with p53 loss in oncogenic transformation. Proc. Natl. Acad. Sci. USA.

[41] Rodriguez-Nieto S., Canada A., Pros E., Pinto A.I., Torres-Lanzas J., Lopez-Rios F., Sanchez-Verde L., Pisano D.G., Sanchez-Cespedes M. (2011). Massive parallel DNA pyrosequencing analysis of the tumor suppressor BRG1/SMARCA4 in lung primary tumors. Hum. Mutat..

[42] Concepcion C.P., Ma S., LaFave L.M., Bhutkar A., Liu M., DeAngelo L.P., Kim J.Y., Del Priore I., Schoenfeld A.J., Miller M. (2022). Smarca4 Inactivation Promotes Lineage-Specific Transformation and Early Metastatic Features in the Lung. Cancer Discov..

[43] Witzel M., Petersheim D., Fan Y., Bahrami E., Racek T., Rohlfs M., Puchalka J., Mertes C., Gagneur J., Ziegenhain C. (2017). Chromatin-remodeling factor SMARCD2 regulates transcriptional networks controlling differentiation of neutrophil granulocytes. Nat. Genet..

[44] Mukherjee S., Stroberg E., Wang F., Morales L., Shan Y., Rao A., Huang J.H., Wu E., Fonkem E. (2020). SMARCB1 Gene Mutation Predisposes to Earlier Development of Glioblastoma: A Case Report of Familial GBM. J. Neuropathol. Exp. Neurol..

[45] Saqcena M., Leandro-Garcia L.J., Maag J.L.V., Tchekmedyian V., Krishnamoorthy G.P., Tamarapu P.P., Tiedje V., Reuter V., Knauf J.A., de Stanchina E. (2021). SWI/SNF Complex Mutations Promote Thyroid Tumor Progression and Insensitivity to Redifferentiation Therapies. Cancer Discov..

[46] Wu J., He K., Zhang Y., Song J., Shi Z., Chen W., Shao Y. (2019). Inactivation of SMARCA2 by promoter hypermethylation drives lung cancer development. Gene.

[47] Smith M.J., Ahn S., Lee J.I., Bulman M., Plessis D.D., Suh Y.L. (2017). SMARCE1 mutation screening in classification of clear cell meningiomas. Histopathology.

[48] Navalkele P., Guzman M., Kirby A., Pinz H., Kemp J. (2020). Familial Pediatric Clear Cell Meningioma With Germline SMARCE1 Mutation in the United States. J. Neuropathol. Exp. Neurol..

[49] Saladi S.V., Ross K., Karaayvaz M., Tata P.R., Mou H., Rajagopal J., Ramaswamy S., Ellisen L.W. (2017). ACTL6A Is Co-Amplified with p63 in Squamous Cell Carcinoma to Drive YAP Activation, Regenerative Proliferation, and Poor Prognosis. Cancer Cell.

[50] Gazendam A.M., Popovic S., Munir S., Parasu N., Wilson D., Ghert M. (2021). Synovial Sarcoma: A Clinical Review. Curr. Oncol..

[51] Mullen J., Kato S., Sicklick J.K., Kurzrock R. (2021). Targeting ARID1A mutations in cancer. Cancer Treat. Rev..

[52] Cajuso T., Hanninen U.A., Kondelin J., Gylfe A.E., Tanskanen T., Katainen R., Pitkanen E., Ristolainen H., Kaasinen E., Taipale M. (2014). Exome sequencing reveals frequent inactivating mutations in ARID1A, ARID1B, ARID2 and ARID4A in microsatellite unstable colorectal cancer. Int. J. Cancer.

[53] Moreno T., Monterde B., Gonzalez-Silva L., Betancor-Fernandez I., Revilla C., Agraz-Doblas A., Freire J., Isidro P., Quevedo L., Blanco R. (2021). ARID2 deficiency promotes tumor progression and is associated with higher sensitivity to chemotherapy in lung cancer. Oncogene.

[54] Zhou H., Liu J., Zhang Y., Huang Y., Shen J., Yang Y., Fang W., Zhang L. (2020). PBRM1 mutation and preliminary response to immune checkpoint blockade treatment in non-small cell lung cancer. NPJ Precis. Oncol..

[55] Sumegi J., Nishio J., Nelson M., Frayer R.W., Perry D., Bridge J.A. (2011). A novel t(4;22)(q31;q12) produces an EWSR1-SMARCA5 fusion in extraskeletal Ewing sarcoma/primitive neuroectodermal tumor. Mod. Pathol..

[56] Wu Y., Ni H., Yang D., Niu Y., Chen K., Xu J., Wang F., Tang S., Shi Y., Zhang H. (2021). Driver and novel genes correlated with metastasis of non-small cell lung cancer: A comprehensive analysis. Pathol. Res. Pract..

[57] Takeshima H., Niwa T., Takahashi T., Wakabayashi M., Yamashita S., Ando T., Inagawa Y., Taniguchi H., Katai H., Sugiyama T. (2015). Frequent involvement of chromatin remodeler alterations in gastric field cancerization. Cancer Lett..

[58] Jin Q., Mao X., Li B., Guan S., Yao F., Jin F. (2015). Overexpression of SMARCA5 correlates with cell proliferation and migration in breast cancer. Tumour Biol..

[59] Gigek C.O., Lisboa L.C., Leal M.F., Silva P.N., Lima E.M., Khayat A.S., Assumpcao P.P., Burbano R.R., Smith Mde A. (2011). SMARCA5 methylation and expression in gastric cancer. Cancer Investig..

[60] Jevtic Z., Matafora V., Casagrande F., Santoro F., Minucci S., Garre M., Rasouli M., Heidenreich O., Musco G., Schwaller J. (2022). SMARCA5 interacts with NUP98-NSD1 oncofusion protein and sustains hematopoietic cells transformation. J. Exp. Clin. Cancer Res. CR.

[61] Shibayama Y., Takahashi K., Yamaguchi H., Yasuda J., Yamazaki D., Rahman A., Fujimori T., Fujisawa Y., Takai S., Furukawa T. (2020). Aberrant (pro)renin receptor expression induces genomic instability in pancreatic ductal adenocarcinoma through upregulation of SMARCA5/SNF2H. Commun. Biol..

[62] Zhang Z., Sang Y., Liu Z., Shao J. (2021). Negative Correlation Between Circular RNA SMARC5 and MicroRNA 432, and Their Clinical Implications in Bladder Cancer Patients. Technol. Cancer Res. Treat..

[63] Xu X., Zhang J., Tian Y., Gao Y., Dong X., Chen W., Yuan X., Yin W., Xu J., Chen K. (2020). CircRNA inhibits DNA damage repair by interacting with host gene. Mol. Cancer.

[64] Burkhardt L., Fuchs S., Krohn A., Masser S., Mader M., Kluth M., Bachmann F., Huland H., Steuber T., Graefen M. (2013). CHD1 is a 5q21 tumor suppressor required for ERG rearrangement in prostate cancer. Cancer Res..

[65] Taylor B.S., Schultz N., Hieronymus H., Gopalan A., Xiao Y., Carver B.S., Arora V.K., Kaushik P., Cerami E., Reva B. (2010). Integrative genomic profiling of human prostate cancer. Cancer Cell.

[66] Li J., Xu C., Lee H.J., Ren S., Zi X., Zhang Z., Wang H., Yu Y., Yang C., Gao X. (2020). A genomic and epigenomic atlas of prostate cancer in Asian populations. Nature.

[67] Zhang Z., Zhou C., Li X., Barnes S.D., Deng S., Hoover E., Chen C.C., Lee Y.S., Zhang Y., Wang C. (2020). Loss of CHD1 Promotes Heterogeneous Mechanisms of Resistance to AR-Targeted Therapy via Chromatin Dysregulation. Cancer Cell.

[68] Laurent C., Nicolae A., Laurent C., Le Bras F., Haioun C., Fataccioli V., Amara N., Adelaide J., Guille A., Schiano J.M. (2020). Gene alterations in epigenetic modifiers and JAK-STAT signaling are frequent in breast implant-associated ALCL. Blood.

[69] Hill H.A., Qi X., Jain P., Nomie K., Wang Y., Zhou S., Wang M.L. (2020). Genetic mutations and features of mantle cell lymphoma: A systematic review and meta-analysis. Blood Adv..

[70] Zhan D., Zhang Y., Xiao P., Zheng X., Ruan M., Zhang J., Chen A., Zou Y., Chen Y., Huang G. (2018). Whole exome sequencing identifies novel mutations of epigenetic regulators in chemorefractory pediatric acute myeloid leukemia. Leuk. Res..

[71] Gao X., Zhang L., Jia Q., Tang L., Guo W., Wang T., Wu Z., Zhou W., Li Z., Xiao J. (2020). Whole Genome Sequencing Identifies Key Genes in Spinal Schwannoma. Front. Genet..

[72] Bagchi A., Papazoglu C., Wu Y., Capurso D., Brodt M., Francis D., Bredel M., Vogel H., Mills A.A. (2007). CHD5 is a tumor suppressor at human 1p36. Cell.

[73] Garcia-Lopez J., Wallace K., Otero J.H., Olsen R., Wang Y.D., Finkelstein D., Gudenas B.L., Rehg J.E., Northcott P., Davidoff A.M. (2020). Large 1p36 Deletions Affecting Arid1a Locus Facilitate Mycn-Driven Oncogenesis in Neuroblastoma. Cell Rep..

[74] Gui Y., Guo G., Huang Y., Hu X., Tang A., Gao S., Wu R., Chen C., Li X., Zhou L. (2011). Frequent mutations of chromatin remodeling genes in transitional cell carcinoma of the bladder. Nat. Genet..

[75] Tahara T., Yamamoto E., Madireddi P., Suzuki H., Maruyama R., Chung W., Garriga J., Jelinek J., Yamano H.O., Sugai T. (2014). Colorectal carcinomas with CpG island methylator phenotype 1 frequently contain mutations in chromatin regulators. Gastroenterology.

[76] Pleasance E.D., Stephens P.J., O’Meara S., McBride D.J., Meynert A., Jones D., Lin M.L., Beare D., Lau K.W., Greenman C. (2010). A small-cell lung cancer genome with complex signatures of tobacco exposure. Nature.

[77] Chu X., Guo X., Jiang Y., Yu H., Liu L., Shan W., Yang Z.Q. (2017). Genotranscriptomic meta-analysis of the CHD family chromatin remodelers in human cancers—Initial evidence of an oncogenic role for CHD7. Mol. Oncol.

[78] Hasan N., Ahuja N. (2019). The Emerging Roles of ATP-Dependent Chromatin Remodeling Complexes in Pancreatic Cancer. Cancers.

[79] Pienkowska-Grela B., Rymkiewicz G., Grygalewicz B., Woroniecka R., Krawczyk P., Czyz-Domanska K., Walewski J. (2011). Partial trisomy 11, dup(11)(q23q13), as a defect characterizing lymphomas with Burkitt pathomorphology without MYC gene rearrangement. Med. Oncol..

[80] Wagener R., Seufert J., Raimondi F., Bens S., Kleinheinz K., Nagel I., Altmuller J., Thiele H., Hubschmann D., Kohler C.W. (2019). The mutational landscape of Burkitt-like lymphoma with 11q aberration is distinct from that of Burkitt lymphoma. Blood.

[81] Soares de Lima Y., Arnau-Collell C., Diaz-Gay M., Bonjoch L., Franch-Exposito S., Munoz J., Moreira L., Ocana T., Cuatrecasas M., Herrera-Pariente C. (2021). Germline and Somatic Whole-Exome Sequencing Identifies New Candidate Genes Involved in Familial Predisposition to Serrated Polyposis Syndrome. Cancers.

[82] McKinney M., Moffitt A.B., Gaulard P., Travert M., De Leval L., Nicolae A., Raffeld M., Jaffe E.S., Pittaluga S., Xi L. (2017). The Genetic Basis of Hepatosplenic T-cell Lymphoma. Cancer Discov..

[83] Lee S.A., Lee H.S., Hur S.K., Kang S.W., Oh G.T., Lee D., Kwon J. (2017). INO80 haploinsufficiency inhibits colon cancer tumorigenesis via replication stress-induced apoptosis. Oncotarget.

[84] Zhang S., Zhou B., Wang L., Li P., Bennett B.D., Snyder R., Garantziotis S., Fargo D.C., Cox A.D., Chen L. (2017). INO80 is required for oncogenic transcription and tumor growth in non-small cell lung cancer. Oncogene.

[85] Zhou B., Wang L., Zhang S., Bennett B.D., He F., Zhang Y., Xiong C., Han L., Diao L., Li P. (2016). INO80 governs superenhancer-mediated oncogenic transcription and tumor growth in melanoma. Genes Dev..

[86] Stopka T., Skoultchi A.I. (2003). The ISWI ATPase Snf2h is required for early mouse development. Proc. Natl. Acad. Sci. USA.

[87] Tyagi M., Imam N., Verma K., Patel A.K. (2016). Chromatin remodelers: We are the drivers!!. Nucleus.

[88] Erdel F., Schubert T., Marth C., Langst G., Rippe K. (2010). Human ISWI chromatin-remodeling complexes sample nucleosomes via transient binding reactions and become immobilized at active sites. Proc. Natl. Acad. Sci. USA.

[89] Li Y., Gong H., Wang P., Zhu Y., Peng H., Cui Y., Li H., Liu J., Wang Z. (2021). The emerging role of ISWI chromatin remodeling complexes in cancer. J. Exp. Clin. Cancer Res. CR.

[90] Yang S., Gao S., Liu T., Liu J., Zheng X., Li Z. (2021). Circular RNA SMARCA5 functions as an anti-tumor candidate in colon cancer by sponging microRNA-552. Cell Cycle.

[91] Alendar A., Berns A. (2021). Sentinels of chromatin: Chromodomain helicase DNA-binding proteins in development and disease. Genes Dev..

[92] Jones D.O., Cowell I.G., Singh P.B. (2000). Mammalian chromodomain proteins: Their role in genome organisation and expression. Bioessays.

[93] Siggens L., Cordeddu L., Ronnerblad M., Lennartsson A., Ekwall K. (2015). Transcription-coupled recruitment of human CHD1 and CHD2 influences chromatin accessibility and histone H3 and H3.3 occupancy at active chromatin regions. Epigenetics Chromatin.

[94] Bergs J.W., Neuendorff N., van der Heijden G., Wassenaar E., Rexin P., Elsasser H.P., Moll R., Baarends W.M., Brehm A. (2014). Differential expression and sex chromosome association of CHD3/4 and CHD5 during spermatogenesis. PLoS ONE.

[95] Sher F., Hossain M., Seruggia D., Schoonenberg V.A.C., Yao Q., Cifani P., Dassama L.M.K., Cole M.A., Ren C., Vinjamur D.S. (2019). Rational targeting of a NuRD subcomplex guided by comprehensive in situ mutagenesis. Nat. Genet..

[96] Ma C., Wang F., Han B., Zhong X., Si F., Ye J., Hsueh E.C., Robbins L., Kiefer S.M., Zhang Y. (2018). SALL1 functions as a tumor suppressor in breast cancer by regulating cancer cell senescence and metastasis through the NuRD complex. Mol. Cancer.

[97] Xia L., Huang W., Bellani M., Seidman M.M., Wu K., Fan D., Nie Y., Cai Y., Zhang Y.W., Yu L.R. (2017). CHD4 Has Oncogenic Functions in Initiating and Maintaining Epigenetic Suppression of Multiple Tumor Suppressor Genes. Cancer Cell.

[98] Daubresse G., Deuring R., Moore L., Papoulas O., Zakrajsek I., Waldrip W.R., Scott M.P., Kennison J.A., Tamkun J.W. (1999). The Drosophila kismet gene is related to chromatin-remodeling factors and is required for both segmentation and segment identity. Development.

[99] Watanabe S., Tan D., Lakshminarasimhan M., Washburn M.P., Hong E.J., Walz T., Peterson C.L. (2015). Structural analyses of the chromatin remodelling enzymes INO80-C and SWR-C. Nat. Commun..

[100] Willhoft O., Wigley D.B. (2020). INO80 and SWR1 complexes: The non-identical twins of chromatin remodelling. Curr. Opin. Struct. Biol..

[101] Shen X., Ranallo R., Choi E., Wu C. (2003). Involvement of actin-related proteins in ATP-dependent chromatin remodeling. Mol. Cell.

[102] Yen K., Vinayachandran V., Pugh B.F. (2013). SWR-C and INO80 chromatin remodelers recognize nucleosome-free regions near +1 nucleosomes. Cell.

[103] Clapier C.R., Cairns B.R. (2009). The biology of chromatin remodeling complexes. Ann. Rev. Biochem..

[104] Cai Y., Jin J., Yao T., Gottschalk A.J., Swanson S.K., Wu S., Shi Y., Washburn M.P., Florens L., Conaway R.C. (2007). YY1 functions with INO80 to activate transcription. Nat. Struct. Mol. Biol..

[105] Uno K., Takita J., Yokomori K., Tanaka Y., Ohta S., Shimada H., Gilles F.H., Sugita K., Abe S., Sako M. (2002). Aberrations of the hSNF5/INI1 gene are restricted to malignant rhabdoid tumors or atypical teratoid/rhabdoid tumors in pediatric solid tumors. Genes Chromosomes Cancer.

[106] Lehmann L.C., Hewitt G., Aibara S., Leitner A., Marklund E., Maslen S.L., Maturi V., Chen Y., van der Spoel D., Skehel J.M. (2017). Mechanistic Insights into Autoinhibition of the Oncogenic Chromatin Remodeler ALC1. Mol. Cell.

[107] Yang Y., Zhao X., Li H.X. (2016). MiR-221 and miR-222 simultaneously target ARID1A and enhance proliferation and invasion of cervical cancer cells. Eur. Rev. Med. Pharmacol. Sci..

[108] Shi Y., Gao S., Zheng Y., Yao M., Ruan F. (2019). LncRNA CASC15 Functions As An Unfavorable Predictor Of Ovarian Cancer Prognosis And Inhibits Tumor Progression Through Regulation Of miR-221/ARID1A Axis. OncoTargets Ther..

[109] Wang L.L., Sun K.X., Wu D.D., Xiu Y.L., Chen X., Chen S., Zong Z.H., Sang X.B., Liu Y., Zhao Y. (2017). DLEU1 contributes to ovarian carcinoma tumourigenesis and development by interacting with miR-490-3p and altering CDK1 expression. J. Cell Mol. Med..

[110] Huang L.Y., Zhao J., Chen H., Wan L., Inuzuka H., Guo J., Fu X., Zhai Y., Lu Z., Wang X. (2018). SCF(FBW7)-mediated degradation of Brg1 suppresses gastric cancer metastasis. Nat. Commun..

[111] Keppler B.R., Archer T.K. (2010). Ubiquitin-dependent and ubiquitin-independent control of subunit stoichiometry in the SWI/SNF complex. J. Biol. Chem..

[112] Morrison A.J., Kim J.A., Person M.D., Highland J., Xiao J., Wehr T.S., Hensley S., Bao Y., Shen J., Collins S.R. (2007). Mec1/Tel1 phosphorylation of the INO80 chromatin remodeling complex influences DNA damage checkpoint responses. Cell.

[113] Zhang L., Li D.Q. (2019). MORC2 regulates DNA damage response through a PARP1-dependent pathway. Nucleic Acids Res..

[114] Liu H.Y., Liu Y.Y., Yang F., Zhang L., Zhang F.L., Hu X., Shao Z.M., Li D.Q. (2020). Acetylation of MORC2 by NAT10 regulates cell-cycle checkpoint control and resistance to DNA-damaging chemotherapy and radiotherapy in breast cancer. Nucleic Acids Res..

[115] Ahel D., Horejsi Z., Wiechens N., Polo S.E., Garcia-Wilson E., Ahel I., Flynn H., Skehel M., West S.C., Jackson S.P. (2009). Poly(ADP-ribose)-dependent regulation of DNA repair by the chromatin remodeling enzyme ALC1. Science.

[116] Verma P., Zhou Y., Cao Z., Deraska P.V., Deb M., Arai E., Li W., Shao Y., Puentes L., Li Y. (2021). ALC1 links chromatin accessibility to PARP inhibitor response in homologous recombination-deficient cells. Nat. Cell Biol..

[117] Juhasz S., Elbakry A., Mathes A., Lobrich M. (2018). ATRX Promotes DNA Repair Synthesis and Sister Chromatid Exchange during Homologous Recombination. Mol. Cell.

[118] Chen Y., Zhang H., Xu Z., Tang H., Geng A., Cai B., Su T., Shi J., Jiang C., Tian X. (2019). A PARP1-BRG1-SIRT1 axis promotes HR repair by reducing nucleosome density at DNA damage sites. Nucleic Acids Res..

[119] Ubhi T., Brown G.W. (2019). Exploiting DNA Replication Stress for Cancer Treatment. Cancer Res..

[120] Thomas A., Takahashi N., Rajapakse V.N., Zhang X., Sun Y., Ceribelli M., Wilson K.M., Zhang Y., Beck E., Sciuto L. (2021). Therapeutic targeting of ATR yields durable regressions in small cell lung cancers with high replication stress. Cancer Cell.

[121] Mognato M., Burdak-Rothkamm S., Rothkamm K. (2021). Interplay between DNA replication stress, chromatin dynamics and DNA-damage response for the maintenance of genome stability. Mutat Res. Rev. Mutat Res..

[122] Landsverk H.B., Sandquist L.E., Bay L.T.E., Steurer B., Campsteijn C., Landsverk O.J.B., Marteijn J.A., Petermann E., Trinkle-Mulcahy L., Syljuasen R.G. (2020). WDR82/PNUTS-PP1 Prevents Transcription-Replication Conflicts by Promoting RNA Polymerase II Degradation on Chromatin. Cell Rep..

[123] Prendergast L., McClurg U.L., Hristova R., Berlinguer-Palmini R., Greener S., Veitch K., Hernandez I., Pasero P., Rico D., Higgins J.M.G. (2020). Resolution of R-loops by INO80 promotes DNA replication and maintains cancer cell proliferation and viability. Nat. Commun..

[124] Tsai S., Fournier L.A., Chang E.Y., Wells J.P., Minaker S.W., Zhu Y.D., Wang A.Y., Wang Y., Huntsman D.G., Stirling P.C. (2021). ARID1A regulates R-loop associated DNA replication stress. PLoS Genet..

[125] Bayona-Feliu A., Barroso S., Munoz S., Aguilera A. (2021). The SWI/SNF chromatin remodeling complex helps resolve R-loop-mediated transcription-replication conflicts. Nat. Genet..

[126] Cox K.E., Marechal A., Flynn R.L. (2016). SMARCAL1 Resolves Replication Stress at ALT Telomeres. Cell Rep..

[127] Davalos A.R., Coppe J.P., Campisi J., Desprez P.Y. (2010). Senescent cells as a source of inflammatory factors for tumor progression. Cancer Metastasis Rev..

[128] Zhang J.W., Zhang D., Yu B.P. (2021). Senescent cells in cancer therapy: Why and how to remove them. Cancer Lett.

[129] Swer P.B., Sharma R. (2021). ATP-dependent chromatin remodelers in ageing and age-related disorders. Biogerontology.

[130] Li X., Ding D., Yao J., Zhou B., Shen T., Qi Y., Ni T., Wei G. (2019). Chromatin remodeling factor BAZ1A regulates cellular senescence in both cancer and normal cells. Life Sci..

[131] Dang W., Sutphin G.L., Dorsey J.A., Otte G.L., Cao K., Perry R.M., Wanat J.J., Saviolaki D., Murakami C.J., Tsuchiyama S. (2014). Inactivation of yeast Isw2 chromatin remodeling enzyme mimics longevity effect of calorie restriction via induction of genotoxic stress response. Cell Metab..

[132] Tordella L., Khan S., Hohmeyer A., Banito A., Klotz S., Raguz S., Martin N., Dhamarlingam G., Carroll T., Gonzalez Meljem J.M. (2016). SWI/SNF regulates a transcriptional program that induces senescence to prevent liver cancer. Genes Dev..

[133] Soshnikova N.V., Tatarskiy E.V., Tatarskiy V.V., Klimenko N.S., Shtil A.A., Nikiforov M.A., Georgieva S.G. (2021). PHF10 subunit of PBAF complex mediates transcriptional activation by MYC. Oncogene.

[134] Liu S., Cao W., Niu Y., Luo J., Zhao Y., Hu Z., Zong C. (2021). Single-PanIN-seq unveils that ARID1A deficiency promotes pancreatic tumorigenesis by attenuating KRAS-induced senescence. eLife.

[135] Oruetxebarria I., Venturini F., Kekarainen T., Houweling A., Zuijderduijn L.M., Mohd-Sarip A., Vries R.G., Hoeben R.C., Verrijzer C.P. (2004). P16INK4a is required for hSNF5 chromatin remodeler-induced cellular senescence in malignant rhabdoid tumor cells. J. Biol. Chem..

[136] Burrows A.E., Smogorzewska A., Elledge S.J. (2010). Polybromo-associated BRG1-associated factor components BRD7 and BAF180 are critical regulators of p53 required for induction of replicative senescence. Proc. Natl. Acad. Sci. USA.

[137] Wang G., Fu Y., Yang X., Luo X., Wang J., Gong J., Hu J. (2016). Brg-1 targeting of novel miR550a-5p/RNF43/Wnt signaling axis regulates colorectal cancer metastasis. Oncogene.

[138] von Figura G., Fukuda A., Roy N., Liku M.E., Morris Iv J.P., Kim G.E., Russ H.A., Firpo M.A., Mulvihill S.J., Dawson D.W. (2014). The chromatin regulator Brg1 suppresses formation of intraductal papillary mucinous neoplasm and pancreatic ductal adenocarcinoma. Nat. Cell Biol..

[139] Roy N., Malik S., Villanueva K.E., Urano A., Lu X., Von Figura G., Seeley E.S., Dawson D.W., Collisson E.A., Hebrok M. (2015). Brg1 promotes both tumor-suppressive and oncogenic activities at distinct stages of pancreatic cancer formation. Genes Dev..

[140] Sun L., Yuan Y., Chen J., Ma C., Xu Y. (2019). Brahma related gene 1 (BRG1) regulates breast cancer cell migration and invasion by activating MUC1 transcription. Biochem. Biophys. Res. Commun..

[141] Yang Y., Liu L., Li M., Cheng X., Fang M., Zeng Q., Xu Y. (2019). The chromatin remodeling protein BRG1 links ELOVL3 trans-activation to prostate cancer metastasis. Biochim. Biophys. Acta Gene Regul. Mech..

[142] Yang Y., Liu L., Fang M., Bai H., Xu Y. (2019). The chromatin remodeling protein BRM regulates the transcription of tight junction proteins: Implication in breast cancer metastasis. Biochim. Biophys. Acta Gene Regul. Mech..

[143] Sun D., Zhu Y., Zhao H., Bian T., Li T., Liu K., Feng L., Li H., Hou H. (2021). Loss of ARID1A expression promotes lung adenocarcinoma metastasis and predicts a poor prognosis. Cell Oncol..

[144] Wang J., Yan H.B., Zhang Q., Liu W.Y., Jiang Y.H., Peng G., Wu F.Z., Liu X., Yang P.Y., Liu F. (2021). Enhancement of E-cadherin expression and processing and driving of cancer cell metastasis by ARID1A deficiency. Oncogene.

[145] Shang X.Y., Shi Y., He D.D., Wang L., Luo Q., Deng C.H., Qu Y.L., Wang N., Han Z.G. (2021). ARID1A deficiency weakens BRG1-RAD21 interaction that jeopardizes chromatin compactness and drives liver cancer cell metastasis. Cell Death Dis..

[146] Sun X., Wang S.C., Wei Y., Luo X., Jia Y., Li L., Gopal P., Zhu M., Nassour I., Chuang J.C. (2017). Arid1a Has Context-Dependent Oncogenic and Tumor Suppressor Functions in Liver Cancer. Cancer Cell.

[147] Jiang H., Cao H.J., Ma N., Bao W.D., Wang J.J., Chen T.W., Zhang E.B., Yuan Y.M., Ni Q.Z., Zhang F.K. (2020). Chromatin remodeling factor ARID2 suppresses hepatocellular carcinoma metastasis via DNMT1-Snail axis. Proc. Natl. Acad. Sci. USA.

[148] Nihan Kilinc A., Sugiyama N., Reddy Kalathur R.K., Antoniadis H., Birogul H., Ishay-Ronen D., George J.T., Levine H., Kumar Jolly M., Christofori G. (2020). Histone deacetylases, Mbd3/NuRD, and Tet2 hydroxylase are crucial regulators of epithelial-mesenchymal plasticity and tumor metastasis. Oncogene.

[149] Chang C.L., Huang C.R., Chang S.J., Wu C.C., Chen H.H., Luo C.W., Yip H.K. (2021). CHD4 as an important mediator in regulating the malignant behaviors of colorectal cancer. Int J. Biol Sci.

[150] Liao X.H., Zhang Y., Dong W.J., Shao Z.M., Li D.Q. (2017). Chromatin remodeling protein MORC2 promotes breast cancer invasion and metastasis through a PRD domain-mediated interaction with CTNND1. Oncotarget.

[151] Zhang F.L., Cao J.L., Xie H.Y., Sun R., Yang L.F., Shao Z.M., Li D.Q. (2018). Cancer-Associated MORC2-Mutant M276I Regulates an hnRNPM-Mediated CD44 Splicing Switch to Promote Invasion and Metastasis in Triple-Negative Breast Cancer. Cancer Res..

[152] Liu Y.Y., Liu H.Y., Yu T.J., Lu Q., Zhang F.L., Liu G.Y., Shao Z.M., Li D.Q. (2022). O-GlcNAcylation of MORC2 at threonine 556 by OGT couples TGF-beta signaling to breast cancer progression. Cell Death Differ..

[153] Teleanu R.I., Chircov C., Grumezescu A.M., Teleanu D.M. (2019). Tumor Angiogenesis and Anti-Angiogenic Strategies for Cancer Treatment. J. Clin. Med..

[154] Zampetaki A., Mayr M. (2017). Long Noncoding RNAs and Angiogenesis: Regulatory Information for Chromatin Remodeling. Circulation.

[155] Liu L., Wan X., Zhou P., Zhou X., Zhang W., Hui X., Yuan X., Ding X., Zhu R., Meng G. (2018). The chromatin remodeling subunit Baf200 promotes normal hematopoiesis and inhibits leukemogenesis. J. Hematol. Oncol..

[156] Hu C., Li W., Tian F., Jiang K., Liu X., Cen J., He Q., Qiu Z., Kienast Y., Wang Z. (2018). Arid1a regulates response to anti-angiogenic therapy in advanced hepatocellular carcinoma. J. Hepatol..

[157] Sethuraman A., Brown M., Seagroves T.N., Wu Z.H., Pfeffer L.M., Fan M. (2016). SMARCE1 regulates metastatic potential of breast cancer cells through the HIF1A/PTK2 pathway. Breast Cancer Res..

[158] Li Q., Shi L., Gui B., Yu W., Wang J., Zhang D., Han X., Yao Z., Shang Y. (2011). Binding of the JmjC demethylase JARID1B to LSD1/NuRD suppresses angiogenesis and metastasis in breast cancer cells by repressing chemokine CCL14. Cancer Res..

[159] Keenan T.E., Burke K.P., van Allen E.M. (2019). Genomic correlates of response to immune checkpoint blockade. Nat. Med..

[160] Zhu G., Shi R., Li Y., Zhang Z., Xu S., Chen C., Cao P., Zhang H., Liu M., Pan Z. (2021). ARID1A, ARID1B, and ARID2 Mutations Serve as Potential Biomarkers for Immune Checkpoint Blockade in Patients With Non-Small Cell Lung Cancer. Front. Immunol..

[161] Li J., Wang W., Zhang Y., Cieslik M., Guo J., Tan M., Green M.D., Wang W., Lin H., Li W. (2020). Epigenetic driver mutations in ARID1A shape cancer immune phenotype and immunotherapy. J. Clin. Investig..

[162] Cai X., Zhou J., Deng J., Chen Z. (2021). Prognostic biomarker SMARCC1 and its association with immune infiltrates in hepatocellular carcinoma. Cancer Cell Int..

[163] Pan D., Kobayashi A., Jiang P., Ferrari de Andrade L., Tay R.E., Luoma A.M., Tsoucas D., Qiu X., Lim K., Rao P. (2018). A major chromatin regulator determines resistance of tumor cells to T cell-mediated killing. Science.

[164] Huang M., Wang H., Hu X., Cao X. (2019). lncRNA MALAT1 binds chromatin remodeling subunit BRG1 to epigenetically promote inflammation-related hepatocellular carcinoma progression. Oncoimmunology.

[165] Malonia S.K., Yadav B., Sinha S., Lazennec G., Chattopadhyay S. (2014). Chromatin remodeling protein SMAR1 regulates NF-kappaB dependent Interleukin-8 transcription in breast cancer. Int. J. Biochem. Cell Biol..

[166] Shao S., Cao H., Wang Z., Zhou D., Wu C., Wang S., Xia D., Zhang D. (2020). CHD4/NuRD complex regulates complement gene expression and correlates with CD8 T cell infiltration in human hepatocellular carcinoma. Clin. Epigenetics.

[167] Yang C., Wang Y., Sims M.M., He Y., Miller D.D., Pfeffer L.M. (2021). Targeting the Bromodomain of BRG-1/BRM Subunit of the SWI/SNF Complex Increases the Anticancer Activity of Temozolomide in Glioblastoma. Pharmaceuticals.

[168] Fedorov O., Castex J., Tallant C., Owen D.R., Martin S., Aldeghi M., Monteiro O., Filippakopoulos P., Picaud S., Trzupek J.D. (2015). Selective targeting of the BRG/PB1 bromodomains impairs embryonic and trophoblast stem cell maintenance. Sci. Adv..

[169] Papillon J.P.N., Nakajima K., Adair C.D., Hempel J., Jouk A.O., Karki R.G., Mathieu S., Mobitz H., Ntaganda R., Smith T. (2018). Discovery of Orally Active Inhibitors of Brahma Homolog (BRM)/SMARCA2 ATPase Activity for the Treatment of Brahma Related Gene 1 (BRG1)/SMARCA4-Mutant Cancers. J. Med. Chem..

[170] Xiao L., Parolia A., Qiao Y., Bawa P., Eyunni S., Mannan R., Carson S.E., Chang Y., Wang X., Zhang Y. (2022). Targeting SWI/SNF ATPases in enhancer-addicted prostate cancer. Nature.

[171] Rago F., DiMare M.T., Elliott G., Ruddy D.A., Sovath S., Kerr G., Bhang H.C., Jagani Z. (2019). Degron mediated BRM/SMARCA2 depletion uncovers novel combination partners for treatment of BRG1/SMARCA4-mutant cancers. Biochem. Biophys. Res. Commun..

[172] Taylor A.M., Bailey C., Belmont L.D., Campbell R., Cantone N., Cote A., Crawford T.D., Cummings R., DeMent K., Duplessis M. (2022). GNE-064: A Potent, Selective, and Orally Bioavailable Chemical Probe for the Bromodomains of SMARCA2 and SMARCA4 and the Fifth Bromodomain of PBRM1. J. Med. Chem..

[173] Melin L., Gesner E., Attwell S., Kharenko O.A., van der Horst E.H., Hansen H.C., Gagnon A. (2021). Design and Synthesis of LM146, a Potent Inhibitor of PB1 with an Improved Selectivity Profile over SMARCA2. ACS Omega.

[174] Clark P.G., Vieira L.C., Tallant C., Fedorov O., Singleton D.C., Rogers C.M., Monteiro O.P., Bennett J.M., Baronio R., Muller S. (2015). LP99: Discovery and Synthesis of the First Selective BRD7/9 Bromodomain Inhibitor. Angew. Chem. Int. Ed. Engl..

[175] Zoppi V., Hughes S.J., Maniaci C., Testa A., Gmaschitz T., Wieshofer C., Koegl M., Riching K.M., Daniels D.L., Spallarossa A. (2019). Iterative Design and Optimization of Initially Inactive Proteolysis Targeting Chimeras (PROTACs) Identify VZ185 as a Potent, Fast, and Selective von Hippel-Lindau (VHL) Based Dual Degrader Probe of BRD9 and BRD7. J. Med. Chem..

[176] Clegg M.A., Bamborough P., Chung C.W., Craggs P.D., Gordon L., Grandi P., Leveridge M., Lindon M., Liwicki G.M., Michon A.M. (2020). Application of Atypical Acetyl-lysine Methyl Mimetics in the Development of Selective Inhibitors of the Bromodomain-Containing Protein 7 (BRD7)/Bromodomain-Containing Protein 9 (BRD9) Bromodomains. J. Med. Chem..

[177] Martin L.J., Koegl M., Bader G., Cockcroft X.L., Fedorov O., Fiegen D., Gerstberger T., Hofmann M.H., Hohmann A.F., Kessler D. (2016). Structure-Based Design of an in Vivo Active Selective BRD9 Inhibitor. J. Med. Chem..

[178] Crawford T.D., Vartanian S., Cote A., Bellon S., Duplessis M., Flynn E.M., Hewitt M., Huang H.R., Kiefer J.R., Murray J. (2017). Inhibition of bromodomain-containing protein 9 for the prevention of epigenetically-defined drug resistance. Bioorg Med. Chem. Lett..

[179] Theodoulou N.H., Bamborough P., Bannister A.J., Becher I., Bit R.A., Che K.H., Chung C.W., Dittmann A., Drewes G., Drewry D.H. (2016). Discovery of I-BRD9, a Selective Cell Active Chemical Probe for Bromodomain Containing Protein 9 Inhibition. J. Med. Chem..

[180] Remillard D., Buckley D.L., Paulk J., Brien G.L., Sonnett M., Seo H.S., Dastjerdi S., Wuhr M., Dhe-Paganon S., Armstrong S.A. (2017). Degradation of the BAF Complex Factor BRD9 by Heterobifunctional Ligands. Angew. Chem. Int. Ed. Engl..

[181] Zahid H., Buchholz C.R., Singh M., Ciccone M.F., Chan A., Nithianantham S., Shi K., Aihara H., Fischer M., Schonbrunn E. (2021). New Design Rules for Developing Potent Cell-Active Inhibitors of the Nucleosome Remodeling Factor (NURF) via BPTF Bromodomain Inhibition. J. Med. Chem..

[182] Vangamudi B., Paul T.A., Shah P.K., Kost-Alimova M., Nottebaum L., Shi X., Zhan Y., Leo E., Mahadeshwar H.S., Protopopov A. (2015). The SMARCA2/4 ATPase Domain Surpasses the Bromodomain as a Drug Target in SWI/SNF-Mutant Cancers: Insights from cDNA Rescue and PFI-3 Inhibitor Studies. Cancer Res..

[183] Rago F., Elliott G., Li A., Sprouffske K., Kerr G., Desplat A., Abramowski D., Chen J.T., Farsidjani A., Xiang K.X. (2020). The Discovery of SWI/SNF Chromatin Remodeling Activity as a Novel and Targetable Dependency in Uveal Melanoma. Mol. Cancer Ther..

[184] Hohmann A.F., Martin L.J., Minder J.L., Roe J.S., Shi J., Steurer S., Bader G., McConnell D., Pearson M., Gerstberger T. (2016). Sensitivity and engineered resistance of myeloid leukemia cells to BRD9 inhibition. Nat. Chem. Biol..

[185] Bekes M., Langley D.R., Crews C.M. (2022). PROTAC targeted protein degraders: The past is prologue. Nat. Rev. Drug Discov..

[186] Schneider M., Radoux C.J., Hercules A., Ochoa D., Dunham I., Zalmas L.P., Hessler G., Ruf S., Shanmugasundaram V., Hann M.M. (2021). The PROTACtable genome. Nat. Rev. Drug Discov..

[187] Farnaby W., Koegl M., Roy M.J., Whitworth C., Diers E., Trainor N., Zollman D., Steurer S., Karolyi-Oezguer J., Riedmueller C. (2019). BAF complex vulnerabilities in cancer demonstrated via structure-based PROTAC design. Nat. Chem. Biol..

[188] Hoffman G.R., Rahal R., Buxton F., Xiang K., McAllister G., Frias E., Bagdasarian L., Huber J., Lindeman A., Chen D. (2014). Functional epigenetics approach identifies BRM/SMARCA2 as a critical synthetic lethal target in BRG1-deficient cancers. Proc. Natl. Acad. Sci. USA.

[189] Mayor-Ruiz C., Bauer S., Brand M., Kozicka Z., Siklos M., Imrichova H., Kaltheuner I.H., Hahn E., Seiler K., Koren A. (2020). Rational discovery of molecular glue degraders via scalable chemical profiling. Nat. Chem. Biol..

[190] Hartwell L.H., Szankasi P., Roberts C.J., Murray A.W., Friend S.H. (1997). Integrating genetic approaches into the discovery of anticancer drugs. Science.

[191] Helming K.C., Wang X., Wilson B.G., Vazquez F., Haswell J.R., Manchester H.E., Kim Y., Kryukov G.V., Ghandi M., Aguirre A.J. (2014). ARID1B is a specific vulnerability in ARID1A-mutant cancers. Nat. Med..

[192] Kelso T.W.R., Porter D.K., Amaral M.L., Shokhirev M.N., Benner C., Hargreaves D.C. (2017). Chromatin accessibility underlies synthetic lethality of SWI/SNF subunits in ARID1A-mutant cancers. eLife.

[193] Shen J., Peng Y., Wei L., Zhang W., Yang L., Lan L., Kapoor P., Ju Z., Mo Q., Shih Ie M. (2015). ARID1A Deficiency Impairs the DNA Damage Checkpoint and Sensitizes Cells to PARP Inhibitors. Cancer Discov..

[194] Park Y., Chui M.H., Suryo Rahmanto Y., Yu Z.C., Shamanna R.A., Bellani M.A., Gaillard S., Ayhan A., Viswanathan A., Seidman M.M. (2019). Loss of ARID1A in Tumor Cells Renders Selective Vulnerability to Combined Ionizing Radiation and PARP Inhibitor Therapy. Clin. Cancer Res..

[195] Chabanon R.M., Morel D., Eychenne T., Colmet-Daage L., Bajrami I., Dorvault N., Garrido M., Meisenberg C., Lamb A., Ngo C. (2021). PBRM1 Deficiency Confers Synthetic Lethality to DNA Repair Inhibitors in Cancer. Cancer Res..

[196] Hagiwara M., Fushimi A., Matsumoto K., Oya M. (2022). The Significance of PARP1 as a biomarker for Predicting the Response to PD-L1 Blockade in Patients with PBRM1-mutated Clear Cell Renal Cell Carcinoma. Eur. Urol..

[197] Hu K., Wu W., Li Y., Lin L., Chen D., Yan H., Xiao X., Chen H., Chen Z., Zhang Y. (2019). Poly(ADP-ribosyl)ation of BRD7 by PARP1 confers resistance to DNA-damaging chemotherapeutic agents. EMBO Rep..

[198] Zhou Q., Huang J., Zhang C., Zhao F., Kim W., Tu X., Zhang Y., Nowsheen S., Zhu Q., Deng M. (2020). The bromodomain containing protein BRD-9 orchestrates RAD51-RAD54 complex formation and regulates homologous recombination-mediated repair. Nat. Commun..

[199] Williamson C.T., Miller R., Pemberton H.N., Jones S.E., Campbell J., Konde A., Badham N., Rafiq R., Brough R., Gulati A. (2016). ATR inhibitors as a synthetic lethal therapy for tumours deficient in ARID1A. Nat. Commun..

[200] Chory E.J., Kirkland J.G., Chang C.Y., D’Andrea V.D., Gourisankar S., Dykhuizen E.C., Crabtree G.R. (2020). Chemical Inhibitors of a Selective SWI/SNF Function Synergize with ATR Inhibition in Cancer Cell Killing. ACS Chem. Biol..

[201] Xue Y., Meehan B., Macdonald E., Venneti S., Wang X.Q.D., Witkowski L., Jelinic P., Kong T., Martinez D., Morin G. (2019). CDK4/6 inhibitors target SMARCA4-determined cyclin D1 deficiency in hypercalcemic small cell carcinoma of the ovary. Nat. Commun..

[202] Geoerger B., Bourdeaut F., DuBois S.G., Fischer M., Geller J.I., Gottardo N.G., Marabelle A., Pearson A.D.J., Modak S., Cash T. (2017). A Phase I Study of the CDK4/6 Inhibitor Ribociclib (LEE011) in Pediatric Patients with Malignant Rhabdoid Tumors, Neuroblastoma, and Other Solid Tumors. Clin. Cancer Res..

[203] Lang J.D., Hendricks W.P.D., Orlando K.A., Yin H., Kiefer J., Ramos P., Sharma R., Pirrotte P., Raupach E.A., Sereduk C. (2018). Ponatinib Shows Potent Antitumor Activity in Small Cell Carcinoma of the Ovary Hypercalcemic Type (SCCOHT) through Multikinase Inhibition. Clin. Cancer Res..

[204] Miller R.E., Brough R., Bajrami I., Williamson C.T., McDade S., Campbell J., Kigozi A., Rafiq R., Pemberton H., Natrajan R. (2016). Synthetic Lethal Targeting of ARID1A-Mutant Ovarian Clear Cell Tumors with Dasatinib. Mol. Cancer Ther..

[205] Wu C., Lyu J., Yang E.J., Liu Y., Zhang B., Shim J.S. (2018). Targeting AURKA-CDC25C axis to induce synthetic lethality in ARID1A-deficient colorectal cancer cells. Nat. Commun..

[206] Mosse Y.P., Fox E., Teachey D.T., Reid J.M., Safgren S.L., Carol H., Lock R.B., Houghton P.J., Smith M.A., Hall D. (2019). A Phase II Study of Alisertib in Children with Recurrent/Refractory Solid Tumors or Leukemia: Children’s Oncology Group Phase I and Pilot Consortium (ADVL0921). Clin. Cancer Res..

[207] Auguste A., Blanc-Durand F., Deloger M., Le Formal A., Bareja R., Wilkes D.C., Richon C., Brunn B., Caron O., Devouassoux-Shisheboran M. (2020). Small Cell Carcinoma of the Ovary, Hypercalcemic Type (SCCOHT) beyond SMARCA4 Mutations: A Comprehensive Genomic Analysis. Cells.

[208] Fukumoto T., Park P.H., Wu S., Fatkhutdinov N., Karakashev S., Nacarelli T., Kossenkov A.V., Speicher D.W., Jean S., Zhang L. (2018). Repurposing Pan-HDAC Inhibitors for ARID1A-Mutated Ovarian Cancer. Cell Rep..

[209] Morel D., Almouzni G., Soria J.C., Postel-Vinay S. (2017). Targeting chromatin defects in selected solid tumors based on oncogene addiction, synthetic lethality and epigenetic antagonism. Ann. Oncol..

[210] Qadeer Z.A., Valle-Garcia D., Hasson D., Sun Z., Cook A., Nguyen C., Soriano A., Ma A., Griffiths L.M., Zeineldin M. (2019). ATRX In-Frame Fusion Neuroblastoma Is Sensitive to EZH2 Inhibition via Modulation of Neuronal Gene Signatures. Cancer Cell.

[211] Zhu Y., Yan C., Wang X., Xu Z., Lv J., Xu X., Yu W., Zhou M., Yue L. (2022). Pan-cancer analysis of ARID family members as novel biomarkers for immune checkpoint inhibitor therapy. Cancer Biol. Ther..

[212] Mishima S., Kawazoe A., Nakamura Y., Sasaki A., Kotani D., Kuboki Y., Bando H., Kojima T., Doi T., Ohtsu A. (2019). Clinicopathological and molecular features of responders to nivolumab for patients with advanced gastric cancer. J. Immunother. Cancer.

[213] Shen J., Ju Z., Zhao W., Wang L., Peng Y., Ge Z., Nagel Z.D., Zou J., Wang C., Kapoor P. (2018). ARID1A deficiency promotes mutability and potentiates therapeutic antitumor immunity unleashed by immune checkpoint blockade. Nat. Med..

[214] Okamura R., Kato S., Lee S., Jimenez R.E., Sicklick J.K., Kurzrock R. (2020). ARID1A alterations function as a biomarker for longer progression-free survival after anti-PD-1/PD-L1 immunotherapy. J. Immunother Cancer.

[215] Kim Y.B., Ahn J.M., Bae W.J., Sung C.O., Lee D. (2019). Functional loss of ARID1A is tightly associated with high PD-L1 expression in gastric cancer. Int. J. Cancer.

[216] Fukumoto T., Fatkhutdinov N., Zundell J.A., Tcyganov E.N., Nacarelli T., Karakashev S., Wu S., Liu Q., Gabrilovich D.I., Zhang R. (2019). HDAC6 Inhibition Synergizes with Anti-PD-L1 Therapy in ARID1A-Inactivated Ovarian Cancer. Cancer Res..

[217] Schoenfeld A.J., Bandlamudi C., Lavery J.A., Montecalvo J., Namakydoust A., Rizvi H., Egger J., Concepcion C.P., Paul S., Arcila M.E. (2020). The Genomic Landscape of SMARCA4 Alterations and Associations with Outcomes in Patients with Lung Cancer. Clin. Cancer Res..

[218] Lissanu Deribe Y., Sun Y., Terranova C., Khan F., Martinez-Ledesma J., Gay J., Gao G., Mullinax R.A., Khor T., Feng N. (2018). Mutations in the SWI/SNF complex induce a targetable dependence on oxidative phosphorylation in lung cancer. Nat. Med..

[219] Bai Y., Xie T., Wang Z., Tong S., Zhao X., Zhao F., Cai J., Wei X., Peng Z., Shen L. (2022). Efficacy and predictive biomarkers of immunotherapy in Epstein-Barr virus-associated gastric cancer. J. Immunother. Cancer..

[220] Kim E.J., Liu P., Zhang S., Donahue K., Wang Y., Schehr J.L., Wolfe S.K., Dickerson A., Lu L., Rui L. (2021). BAF155 methylation drives metastasis by hijacking super-enhancers and subverting anti-tumor immunity. Nucleic Acids Res..

[221] Menasche B.L., Davis E.M., Wang S., Ouyang Y., Li S., Yu H., Shen J. (2020). PBRM1 and the glycosylphosphatidylinositol biosynthetic pathway promote tumor killing mediated by MHC-unrestricted cytotoxic lymphocytes. Sci. Adv..

[222] Miao D., Margolis C.A., Gao W., Voss M.H., Li W., Martini D.J., Norton C., Bosse D., Wankowicz S.M., Cullen D. (2018). Genomic correlates of response to immune checkpoint therapies in clear cell renal cell carcinoma. Science.

[223] Calagua C., Ficial M., Jansen C.S., Hirz T., Del Balzo L., Wilkinson S., Lake R., Ku A.T., Voznesensky O., Sykes D.B. (2021). A Subset of Localized Prostate Cancer Displays an Immunogenic Phenotype Associated with Losses of Key Tumor Suppressor Genes. Clin. Cancer Res..

